# Emergent decision-making behaviour and rhythm generation in a computational model of the ventromedial nucleus of the hypothalamus

**DOI:** 10.1371/journal.pcbi.1007092

**Published:** 2019-06-03

**Authors:** Duncan J. MacGregor, Gareth Leng

**Affiliations:** Centre for Discovery Brain Sciences, University of Edinburgh, Edinburgh, United Kingdom; Norwegian University of Life Sciences, NORWAY

## Abstract

The ventromedial nucleus of the hypothalamus (VMN) has an important role in diverse behaviours. The common involvement in these of sex steroids, nutritionally-related signals, and emotional inputs from other brain areas, suggests that, at any given time, its output is in one of a discrete number of possible states corresponding to discrete motivational drives. Here we explored how networks of VMN neurons might generate such a decision-making architecture. We began with minimalist assumptions about the intrinsic properties of VMN neurons inferred from electrophysiological recordings of these neurons in rats *in vivo*, using an integrate-and-fire based model modified to simulate activity-dependent post-spike changes in neuronal excitability. We used a genetic algorithm based method to fit model parameters to the statistical features of spike patterning in each cell. The spike patterns in both recorded cells and model cells were assessed by analysis of interspike interval distributions and of the index of dispersion of firing rate over different binwidths. Simpler patterned cells could be closely matched by single neuron models incorporating a hyperpolarising afterpotential and either a slow afterhyperpolarisation or a depolarising afterpotential, but many others could not. We then constructed network models with the challenge of explaining the more complex patterns. We assumed that neurons of a given type (with heterogeneity introduced by independently random patterns of external input) were mutually interconnected at random by excitatory synaptic connections (with a variable delay and a random chance of failure). Simple network models of one or two cell types were able to explain the more complex patterns. We then explored the information processing features of such networks that might be relevant for a decision-making network. We concluded that rhythm generation (in the slow theta range) and bistability arise as emergent properties of networks of heterogeneous VMN neurons.

## Author summary

When the needs of an animal require the execution of particular behaviours, the brain must decide which of these needs to prioritise–whether to flee from or fight an aggressor for example, or whether to hunt for food or pursue sex. The ventromedial nucleus of the hypothalamus is involved in such decisions, in the regulation of aggression, feeding behaviour and sexual behaviour. We began with evidence of the electrical activity patterns of these neurons, and from evidence of how they are interconnected. We built computational models to understand how the activity patterns could arise from mutually excitatory connections amongst simple neuron “types”. With a network framework that could explain the observed patterns, we asked what information processing features of such networks might be relevant for decision-making. Two important features arise as emergent properties of such networks; slow oscillatory rhythms–a phenomenon believed to be important for co-ordinating activity in different brain regions–and bistability. A bistable network has an upstate where neurons are active (and, in this case, generating a coherent rhythm) and a downstate, where neurons are inactive. Alternation between these states, we propose, reflects a switch between different behavioural states.

## Introduction

The ventromedial nucleus of the hypothalamus (VMN) is a large hypothalamic nucleus with an important role in diverse behaviours stretching beyond its classic role in appetite regulation and energy homeostasis [1]. The nucleus regulates glucose and lipid homeostasis [2–4], appetite and energy expenditure [5–8]; but also sexual behaviour [9–11], social behaviours and aggression [12,13], and defensive and escape behaviours [14–16]. The diversity of functions regulated by the VMN and the common involvement in these of sex steroids, nutritionally-related signals, and emotional inputs from other brain areas, has led to the suggestion that subpopulations of VMN neurons “constitute a nutritionally sensitive switch, modulating the competing motivations of feeding and avoidance of potentially dangerous environments” [17]. It has similarly been suggested that a ‘switch’ in the VMN might underlie the reciprocal gating of sexual and feeding behaviour [18]. This suggests that the VMN is a multi-stable network–that, at any given time, its output is in one of a discrete number of possible states corresponding to discrete motivational drives.

How might this behaviour arise in the neuronal networks within the VMN? The neurons of this nucleus are densely interconnected, and the great majority of them are glutamatergic: mRNA for the vesicle glutamate transporter VGLUT2 is densely expressed throughout the VMN, while the VMN is virtually devoid of GAD65 and GAD67 mRNA, indicating that it contains few intrinsic GABA neurons [19]. This suggests that VMN neurons are extensively interconnected by mutually excitatory pathways.

For mutual excitation to support stable firing in a subpopulation of neurons, such positive feedback must be restrained by activity-dependent inhibition. In the case of VMN neurons, there is good reason to think that activity-dependent inhibition arises from intrinsic neuronal mechanisms–from slow, spike-dependent hyperpolarising currents. A conspicuous feature of many VMN neurons is that, *in vivo*, spikes are followed by a prolonged relative refractory period, as evidenced by spontaneous spike patterning [20]. Other neurons have a brief refractory period followed by a brief period of hyperexcitability. Such spike-dependent hyperexcitability might arise either by intrinsic mechanisms (depolarising after-potentials (DAPs) that can arise by multiple mechanisms over different time scales), or by recurrent excitatory pathways.

Detailed analysis of spiking activity in neurons of the VMN *in vivo* [20] previously detected a number of distinctive electrophysiological “phenotypes”–consistent patterns of spiking activity that could be classified into about nine behavioural types. These types vary in complexity: some cells show apparently random spike intervals, shaped only by an initial refractory period, while others show short rapid bursts of spikes, or underlying oscillatory activity. Here we used modelling to explore what combinations of distinct cell properties and network connectivity might explain the heterogeneity observed in the VMN.

We modelled single neurons of the VMN using an integrate-and-fire based model with post-spike excitability modified by simplified afterpotentials. We have previously used this approach to model hypothalamic oxytocin and vasopressin neurons: these have no direct or indirect, synaptic interactions between them, so we can directly infer intrinsic properties from their spiking patterns. We found that a model neuron with two spike-dependent mechanisms mimicking a large, brief post-spike hyperpolarisation (a hyperpolarising afterpotential, HAP) and a small but prolonged post-spike afterhyperpolarisation (AHP) can very closely match the spike patterning of oxytocin cells *in vivo* [21,22] and that these simplified activity-dependent potentials are consistent with a biophysically detailed Hodgkin-Huxley type model of those neurons [23]. In modelling vasopressin cells, a close quantitative match to their more complex phasic burst spiking patterns could be achieved with the addition of a DAP and a spike-suppressed hyperpolarisation, acting together to produce emergent bistability, and resulting in intrinsic bursting activity [24].

In the VMN, however, spiking patterns are the product of intrinsic mechanisms combined with network interactions. To model VMN neurons therefore requires assembling a network, but the intrinsic properties of the neurons comprising that network cannot be inferred directly from their observed spiking patterns. Accordingly, we began with minimalist assumptions about their intrinsic properties as inferred from studies of these neurons *in vivo*, using an integrate-and-fire based model as a flexible template constrained to be consistent with experimental observations. We then constructed network models with the challenge of finding simple explanations of those patterns. Finally, with a network model framework that seemed able to explain most observed patterns, we asked what information processing features of such networks are likely to be relevant for a decision-making network.

The recorded VMN spike data, model source code, and software, compiled for Windows PC, are available at https://github.com/HypoModel/VMNNet/releases.

## Results

We used a library of extracellular recordings from 271 neurons of the VMN of anaesthetised male rats [20]. These comprised time series of spike events collected with a temporal resolution of 0.1 ms from recording periods of 15–60 min. In the original publication of these data, the neurons were classified into types from clusters of approximately equal size displaying different spike patterning, detected by analysis of hazard functions constructed from the interspike intervals (ISIs). We selected extracts of five recordings from each type for further analysis and for fitting with single neuron models. The neurons were chosen to represent the range of patterning within each cluster, and extracts were chosen that showed apparent stationarity, as assessed by a relatively constant minute-by-minute level of activity.

**Fig 1:**
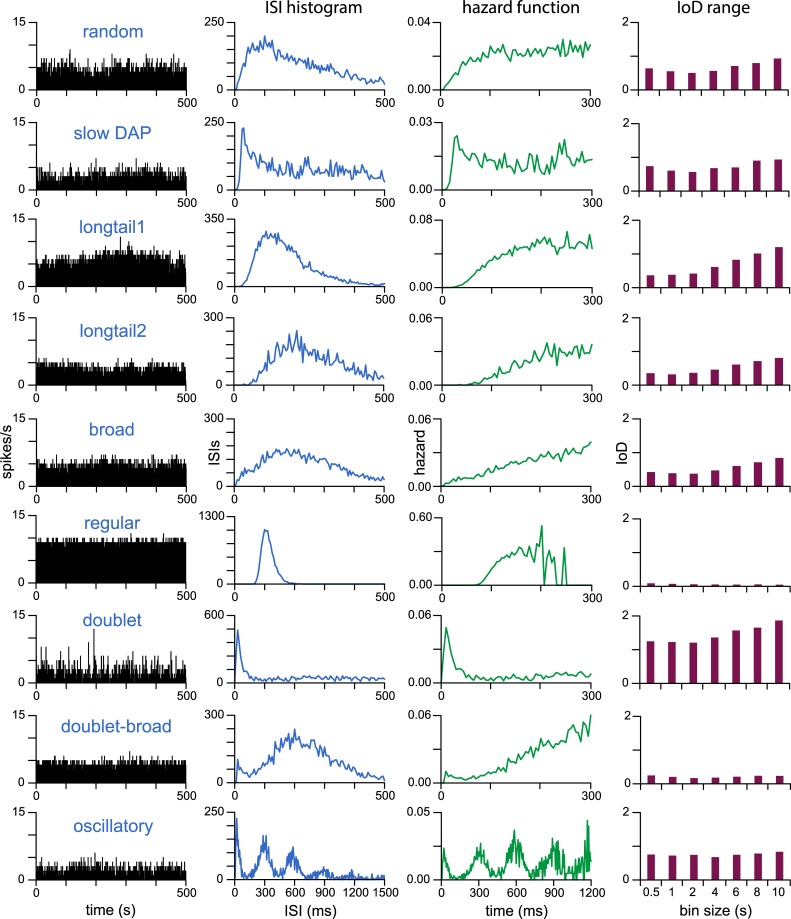
Neural subtypes in the VMN identified by spike patterning. Typical examples of each cell type previously identified [20] from 271 *in vivo* extracellular recordings of the VMN. The left column shows extracts of firing rate in 1-s bins, to the right of these are the corresponding ISI distribution constructed in 10-ms bins over 500 s of spontaneous activity, the corresponding hazard function, and the IoD range. Cells were classified on the basis of ISI analysis. “random”, “slow DAP”, “longtail” (type 1 and 2), and “broad” cells show mostly random patterning subject to varying lengths of post-spike refractory period. “Regular” cells show highly regular spike intervals. “doublet”, “doublet-broad”, and “oscillatory” cells show more complex patterning including short bursts and bimodal ISI histograms. The oscillatory cells have multimodal ISI distributions. Many cells show an increasing IoD range, indicating either a noisy input signal or some positive feedback mechanism, generated either by an intrinsic post-spike depolarisation or network mutual excitation.

For each extract we constructed the ISI distribution in 5-ms bins and the corresponding hazard function, and calculated the index of dispersion (IoD) of firing rate across bin widths of 0.5,1,2,4,6,8 and 10s (IoD range) (Fig 1). The hazard function transforms the ISI distribution to display how the excitability of a neuron changes with time after a spike. The IoD of firing rate, calculated at multiple bin widths, gives a measure of the variability of a neuron’s firing rate and how this depends on the timescale over which the firing rate is measured. Purely random spiking will produce an IoD of 1 independent of the timescale. Regular spiking activity will produce an IoD of <1, while clustered spiking activity will have an IoD > 1 that strongly depends on the timescale. This measure of variability is much more sensitive to slow activity-dependent influences on spike activity than the ISI distribution or hazard function. In oxytocin cells, a slow AHP causes the IoD to reduce as the binwidth increases [21], while a slow DAP can have the opposite effect.

Our neural model is based on the leaky integrate-and-fire model enhanced by the addition of post-spike potentials; it is similar to the ‘spike response’ model [25] but retains a differential equation based form. Activity-dependent potentials, in the form of the HAP, AHP, and DAP, are simulated by spike-triggered step changes in membrane potential followed by exponential decay. In contrast to the classic integrate-and-fire model, there is no post-spike reset of the variables, allowing the DAP and AHP in particular, with their longer half-lives, to accumulate across multiple ISIs. We have previously shown that this modelling of post-spike potentials can be closely mapped to detailed biophysical models, matching the time course and spike patterning effects of voltage- and Ca^2+^- activated ionic conductances [23]. The model’s parameters are based on our previous model of oxytocin neurons [21], which shows similar spike rates and patterning to the “random” type VMN neurons. Oxytocin neurons have been studied in great detail both *in vitro* and *in vivo* and we have good quantitative measures of parameters such as resting potential and spiking threshold, and magnitude of potentials such as the HAP and AHP. We don’t have the same data for VMN neurons but they are likely to be similar and we know from modelling the oxytocin neurons that the model’s behaviour is not dependent on precise values for these parameters [21].

The output of the model is a series of spike times that can be analysed in the same way as recorded spike data. Fitting the model involves identifying values for parameters corresponding to the mean synaptic input rate and the half-lives and magnitudes of the post-spike potentials. We assume that synaptic input is a mixture of a fixed ratio of excitatory postsynaptic potentials (EPSPs) and inhibitory postsynaptic potentials (IPSPs) of fixed magnitude and half-life, so only a single parameter *I*_*re*_ defines the synaptic input. The best fit was determined by comparing the ISI distribution, hazard function, and IoD values between model output and target data.

### VMN cell types determined by the intrinsic neuron parameters

Using two post-spike potentials the model has 11 parameters, but only five of these are required to fit the model to data from a given VMN cell (one for input rate, two for the HAP, and two for the optional AHP or DAP) making it amenable to an automated fitting procedure based on a genetic algorithm (GA), and enabling a more objective and thorough exploration of the parameter space. The GA based technique involves generating a population of random parameter sets, using each set to run the model and generate a fit score, and then ‘evolving’ these over a number of generations to find a best fit. For each cell, we fitted the model (default parameters in Table 1) using a population size of 128 parameter sets in each generation; this was run for 40 generations, varying the parameters within a physiologically plausible range (Table 2). We have previously demonstrated with the same model and fit scoring that this is sufficient to make a robust exploration of the parameter space [23].

**Table 1:** Single neuron model default parameters.

Name	Description	Value	Units
*I*_re_	external input rate	300	Hz
*I*_ratio_	inhibitory input ratio	1	-
*e*_h_	EPSP amplitude	3	mV
*i*_h_	IPSP amplitude	-3	mV
λ_*syn*_	PSP half life	7.5	ms
*k*_*HAP*_	HAP amplitude per spike	30	mV
λ_*HAP*_	HAP half life	8	ms
*k*_*AHP*_	AHP amplitude per spike	0	mV
λ_*AHP*_	AHP half life	500	ms
*k*_*DAP*_	DAP amplitude per spike	0	mV
λ_*DAP*_	DAP half life	1000	ms
*V*_rest_	resting potential	-62	mV
*V*_thresh_	spike threshold potential	-50	mV

**Table 2:** Single neuron model fitting parameter ranges.

Name	Min Value	Max Value
*I*_re_	100	2000
*k*_*HAP*_	0	100
λ_*HAP*_	2	100
*k*_*AHP*_	0	5
λ_*AHP*_	50	1500
*k*_*DAP*_	0	10
λ_*DAP*_	20	2000

To choose a final fit for each cell, we ran the algorithm 100 times and chose the final parameter values as the median values from the 10 best fits. The best fits showed little variation in parameter values, giving confidence in the final fit parameters. For each cell we repeated this process using just an HAP, an HAP and an AHP, and an HAP and a DAP. We also tested the combination of HAP, AHP, and DAP but did not find any cells where the fit could be substantially improved by using all three.

**Fig 2:**
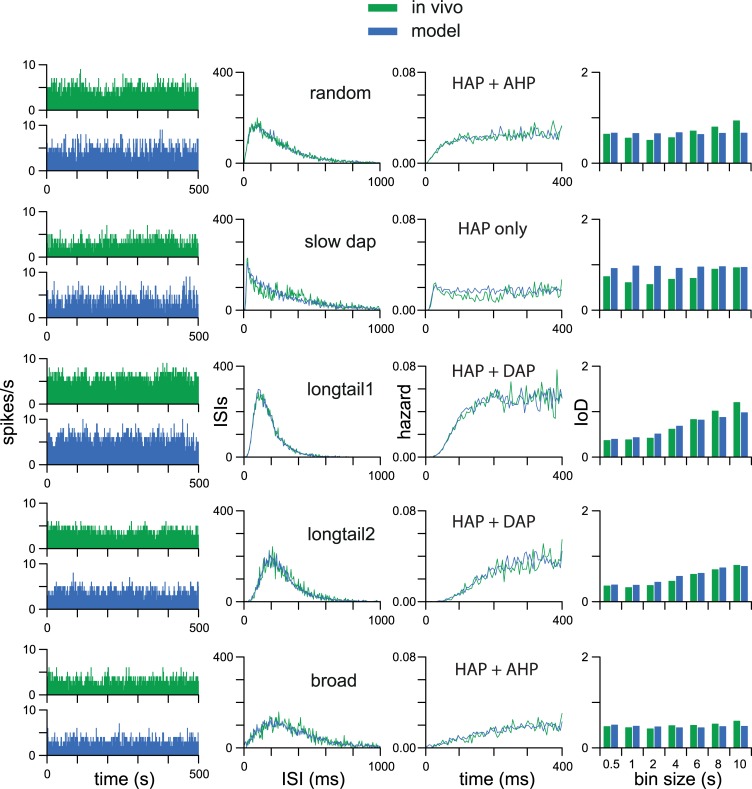
Single neuron model fits. Using automated fitting we produced close matches to the spike patterning of “random”, “slow DAP”, “longtail1”, “longtail2”, and “broad” type VMN neurons, using a single neuron integrate-and-fire based model receiving random synaptic input at a fixed rate. Each row shows a single neuron and its best model fit. The 1-s binned spike rate (1st column) shows the match to spiking rate and variability but not detailed patterning. The fit is measured using a weighted sum of the match to the ISI distribution (2nd column), the hazard function (3rd column), and the IoD range (4th column). The best fits use either just an HAP, an HAP and an AHP, or an HAP and a DAP, indicated in the 3rd column. The early mode in the ISI distribution of the “random” and “slow DAP” type neurons indicates a short refractory period, corresponding with a faster (shorter half-life) HAP fit than in the “longtail1”, “longtail2”, and “broad” type neurons.

Of the nine cell types recognised previously, five types could be well-fitted by the single neuron model (Fig 2 and S1–S5 Figs). Four of the five cells originally classified as “random cells” [20] were fitted by a fast HAP (mean λ_HAP_ = 9.1 ms) and a short AHP (mean λ_AHP_ = 92 ms); the fifth required a DAP for a good fit, to match high IoD values. Four other cell types (“slow DAP”, “longtail1”, “longtail2”, and “broad”) could also all be well fitted with the model, and the fit scores and parameters for all 25 cells are given in S1 Table. Fig 2 shows examples of the fits achieved for one cell of each of these five types, and the fits for all cells are shown in S1 to S5 Figs.

The “slow DAP” cells have a peak in their hazard function that was previously attributed to a DAP. However, this could equally arise from a very fast HAP (mean λ_HAP_ = 4.7 ms): if λ_HAP_ is less than the PSP half-life (7.5 ms) then the accumulated EPSPs that have triggered the spike can have a depolarising effect that outlasts the HAP [26]. Only one of the “slow DAP” cells needed a DAP for the best fit.

The two classes of “longtail” cells could all be fitted with a slow HAP (“longtail1” mean λ_HAP_ = 39 ms and “longtail2” mean λ_HAP_ = 57 ms). Most also required a small DAP for their best fit, with none needing an AHP. The “broad” cells are characterised by a hazard function which has a very slowly decaying refractory period. This was fitted by a fairly slow but small HAP (mean λ_HAP_ = 22 ms; mean *k*_HAP_ = 20 mV).

**Fig 3:**
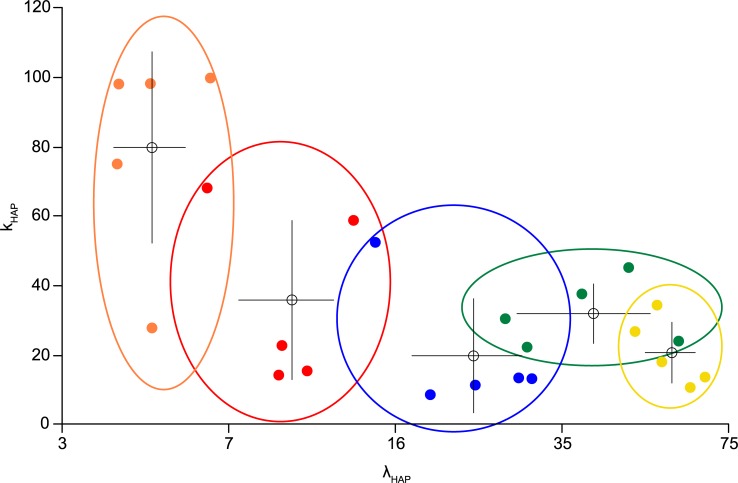
HAP parameter range across cell types and single neuron fits. Each cloud shows five sample fits (each dot a fit) colour coded according to the previously classified VMN cell types (Fig 2). Plotting fits using the HAP fit parameters (*k*_*HAP*_ and λ_*HAP*_), defining the HAP magnitude and half-life, shows a good correlation with cell type. The x-axis is plotted on a natural log scale. The coloured dots show individual cell fits. The white dots and crosses show the mean and standard deviations. The cloud ovals show the range. Longer half-life values tend to correlate with a smaller magnitude. The clouds show overlap, but overall the results are consistent with previous classification, and consistent with the inference that the dominant intrinsic property which differentiates spike patterning across these types is the HAP.

The most important factors in distinguishing between these cell types were the HAP parameters (Fig 2), and the ovals in Fig 3 illustrate the range for each cell type. The previous type classifications remained robust on the basis of model fitting but show some overlap in HAP parameters. The duration of the apparent relative refractory period observed in hazard functions mainly reflects the combined effects of the HAP magnitude and half-life. We estimated this duration as the time taken for the membrane potential of a model cell to return to within 1 mV of the resting potential after a spike in the absence of any synaptic input. The “random” cells and “slow DAP” cells have relative refractory periods of 48 ms and 31 ms respectively (estimated from median parameter values for the cluster); the “longtail1” and “longtail2” cells have relative refractory periods of 198 and 293 ms; and the “broad” cells a relative refractory period of 96 ms.

**Fig 4:**
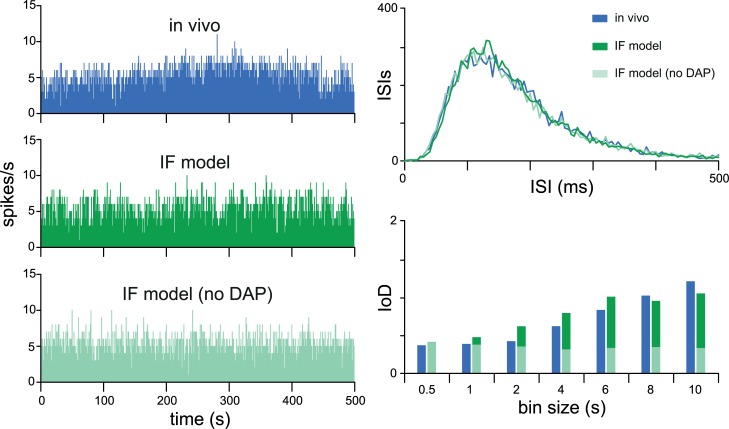
Using a DAP to fit an increasing IoD range. Noise or variability in the spike rate is measured using the IoD. The presence of a large AHP results in a decreasing IoD range, where variability is less at larger binwidths [21]. An increasing IoD range can be mimicked either by a highly variable input signal, or by the amplification of variability due to the action of a DAP. The green *in vivo* example here is a “random” type cell which shows an increasing IoD range. The ISI distribution can be closely matched by a model neuron with only an HAP (red data), but to also fit the increasing IoD range requires a model neuron with a DAP as well as an HAP (blue data).

Four of the five “broad” cells needed an AHP as well as an HAP to achieve the best fit, while six of the ten “longtail” cells needed a small but slow DAP as well as an HAP. In neurons with an AHP, the IoD decreases with increasing binwidth, as previously observed in oxytocin neurons [21,23]. The slow DAP has the opposite effect, producing a higher IoD with longer binwidths (Fig 4). Essentially, a slow DAP makes cells more ‘bursty’ by its positive feedback effects, while a slow AHP makes them more stable by its negative-feedback effects. Thus requiring model cells to fit IoD range data ensures that they capture these features of recorded neurons.

The sixth cell type, “regular cells”, have a hazard function that rises monotonically to very high values and have a very low IoD. This is consistent with a resting potential which lies above the spiking threshold, and a slowly decaying post-spike hyperpolarization that acts as a pacemaker current. However, as we see below, this is not the only way that such patterning can arise.

We conclude that a substantial portion of the heterogeneity in the spike patterning of VMN neurons is due to heterogeneity in their intrinsic mechanisms that shape post-spike excitability. The dominant effect of these is activity-dependent inhibition, and under this there are two substantial subpopulations (Fig 3), distinguished by differences in the duration of post-spike hyperpolarisation. The “random” and “slow DAP” cells displayed a relatively brief hyperpolarisation whereas the “longtail1”, “longtail2”, and “broad” cells displayed a long hyperpolarisation, and hereafter we call these consolidated types ‘fast HAP’ and ‘slow HAP’ cells respectively.

Fitting these cells with a single neuron model subject to random synaptic input shows that the spike patterning in these cell types can be explained purely by intrinsic properties that shape post-spike excitability, using known mechanisms such as the HAP and DAP. It does not assume that they are disconnected from other neurons but rather that there is no coordinated patterning or feedback in the inputs they are receiving. The more complex patterned cell types which could not be fitted with the single neuron model indicate either some unknown intrinsic mechanism or some non-random structure in their inputs. Our subsequent studies tested the idea that this might be the consequence of local network interactions.

### VMN cell types determined by network interactions

Three VMN cell types—those originally classified as “doublet”, “doublet-broad”, and “oscillatory” cells—have multi-modal ISI histograms and hazard functions that we could not match with the single neuron model. To test the idea that these might be network generated, and based on the evidence that most of the VMN neurons are glutamatergic [19], we began by constructing a network of neurons connected by excitatory synapses.

**Fig 5:**
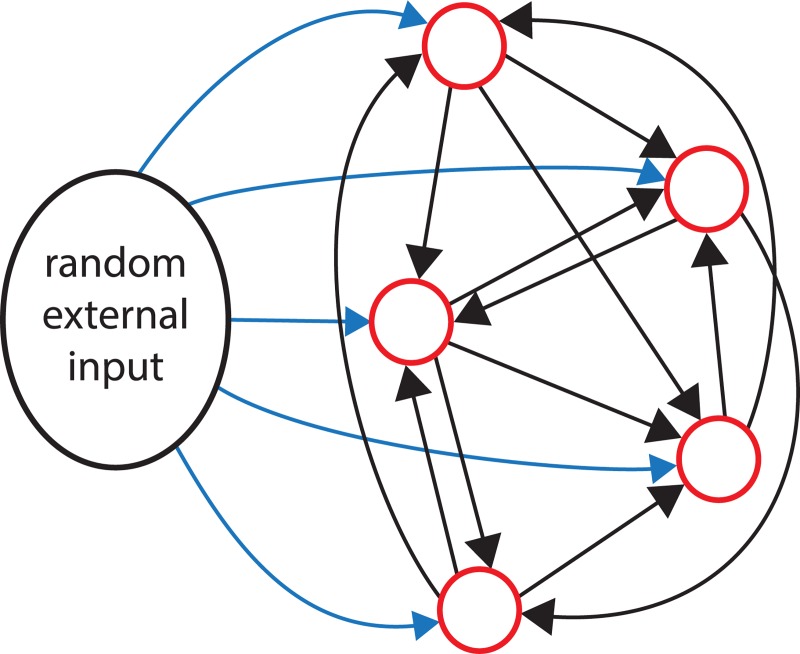
Single cell-type excitatory network. In this network model, identical model neurons are randomly connected, with a chance of connection from one neuron to another defined by *esyn*_*1*_. Connection can independently go in both directions. Network synaptic transmission is modelled by a single spike generating a single EPSP in connected neurons, subject to random transmission failure (0.5 chance), and fixed random transmission delay within a defined range, usually 5–15 ms. Neurons also receive randomly timed external synaptic input at a defined rate, of mixed EPSPs and IPSPs.

The simplest cell types, those that could be well fit by the fast HAP and slow HAP single cell models, defined the building blocks for the subsequent network models. For initial testing we constructed networks of 50 model neurons (Fig 5) with identical intrinsic parameters, based on ‘fast HAP’ cells (*k*_*HAP*_ = 40, λ_*HAP*_ = 10) or ‘slow HAP’ cells (*k*_*HAP*_ = 20, λ_*HAP*_ = 40), both with no AHP or DAP (parameters in Table 3). We used a simple model of synaptic transmission where a single spike generates a single EPSP, of fixed amplitude, subject to a transmission delay. Summation of PSPs within a single time step is linear, although non-linearity is introduced by their exponential decay. The network is randomly generated, with any two neurons having a chance of connecting defined by parameter *esyn*_*1*_. The connections have a fixed strength defined by parameter *synweight* which is used to modify the magnitude of PSPs triggered by spikes generated within the network.

**Table 3:** Single cell type network model parameters (others as in Table 1).

Name	Description	Value	Units
*vmn1*	number of type 1 neurons	50	-
*I*_re_	external input rate	200	Hz
*I*_ratio_	inhibitory input ratio	0.5	-
*k*_*HAP*_ (fast)	HAP amplitude per spike	40	mV
λ_*HAP*_ (fast)	HAP half life	10	ms
*k*_*HAP*_ (slow)	HAP amplitude per spike	20	mV
λ_*HAP*_ (slow)	HAP half life	40	ms
*esyn*_*1*_	type 1 to type1 connection probability	0.5	-
*synweight_1_*	network PSP weighting	1	-
*syntrans*	synaptic transmission probability	0.5	-
*Δ*_*min*_	transmission delay minimum	5	ms
*Δ*_*range*_	transmission delay random component	10	ms
*k*_*syn*_	network PSP magnitude	3	mV
λ_*syn*_	network PSP half-life	7.5	ms

Such networks tended to over-synchronise, shifting suddenly from slow spiking to fast synchronised bursts. We therefore increased the noise and variation in the synaptic connectivity by adding a random chance of failure to synaptic transmission (fixed at probability 0.5) and a variable transmission delay. This produced a more gradual evolution of spike patterning as the synaptic connectivity was progressively increased. In the VMN there are likely to be varied transmission delays between neurons, depending on varied propagation delays, dendritic tree structures, and synapse locations, although how much they might vary is very hard to estimate. To test this assumption we went back to our experimental data to find paired recordings of coupled VMN cells that could be analysed to measure their coupling latency (S6 Fig). The two pairs shown in this Figure show latencies consistent with transmission delays in the range of 5 to 15 ms.

**Fig 6:**
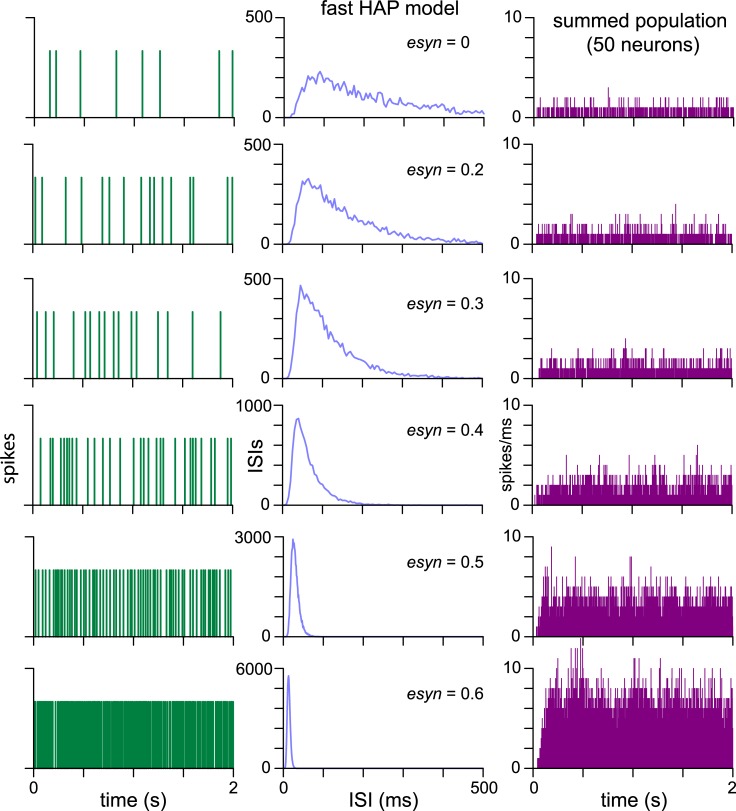
Increasing connection density in an excitatory ‘fast HAP’ network. Each row illustrates a single network model of 50 slow HAP neurons, with connection probability (*esyn*_*1*_) increasing down the rows. All other neuron model parameters are fixed. In each row, the first and second columns show the spiking and ISI distribution for one neuron in the network. Neurons within each network show very similar patterning, with some small variation due to the random connections and random external input signal. The third column shows the summed activity of all neurons in the network. As *esyn*_*1*_ increases, the spike rate increases, the ISI distribution becomes less skewed and the amplitude of the mode increases, but there is otherwise no change in spike patterning. At higher connectivity the summed population activity (right column) shows the shift from slow to fast spiking sustained by the network.

**Fig 7:**
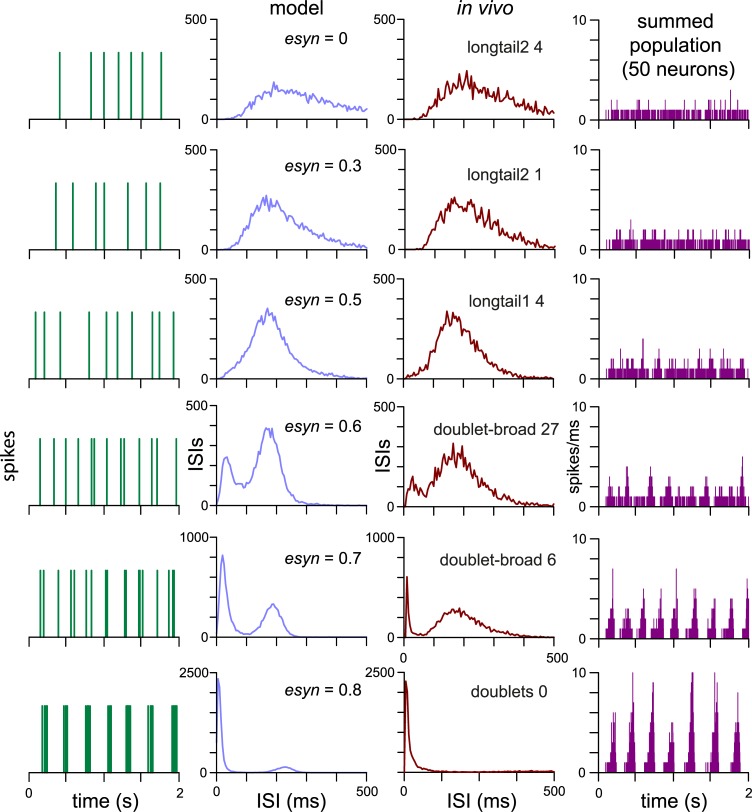
Increasing connection density in an excitatory ‘slow HAP’ network. Each row illustrates a single network model of 50 slow HAP neurons, with connection probability (*esyn*_*1*_) increasing down the rows. All other neuron model parameters are fixed. The example data for each network (1st and 2nd column) shows the spiking and ISI distribution for a typical single neuron. Neurons within each network model show very similar patterning, with some small variation due to the random connections and random external input signal. The *in vivo* column shows recorded VMN cells which have not been directly fitted, but which show patterning very similar to those of neurons in the corresponding network model. As *esyn*_*1*_ increases, the ISI distribution becomes less skewed and the amplitude of the mode increases until a second mode appears, accompanied by the appearance of short bursts. As the second ISI mode grows and dominates, the bursts become faster and more regular, matching “doublet” type VMN cells. The population column (far right) shows the summed spiking activity of the 50 neurons. The noisy synchronisation of the network produces conical peaks that form oscillations.

The ‘fast HAP’ network, subject to the same random input signal, shows a gradual shift to much faster more regular spiking as the connectivity increases (Fig 6). There are no new matches to other types of VMN neurons. However, in the ‘slow HAP’ network, as the connectivity increases, the firing rate increases, and the ISI distributions of individual cells become less skewed until they develop a second mode, matching the spiking observed in “doublet” neurons (Fig 7). With further increases in connectivity, these neurons display regular short bursts of spikes. The change in patterning appears to depend on the combination of intrinsic properties and network connectivity, with the slow HAP forming a negative feedback to counter the positive feedback of the excitatory connections.

The progressive changes in the ISI distribution with increasing *esyn*_*1*_ closely correspond to the ISI distributions of several VMN cell types (Fig 7). Thus, a fixed slow HAP neuron model with a single parameter change increasing network connectivity is sufficient to reproduce much of the heterogeneity observed in VMN spiking patterns.

### Network model fitting

We further tested the ability of the network model to match VMN cell patterning, attempting precise fits to five sample cells from the “doublet” and “doublet-broad” cell types. We attempted to adapt the automated fitting to the network model with a network version, but this was not sufficiently robust in the quality and consistency of the fits it produced. However, using manual parameter adjustment, informed by the matches observed in Fig 7, we could achieve close fits with 50-neuron network models to both the “doublet” and “doublet-broad” type neurons (S7 and S8 Figs; parameters in S2 Table).

The fits require the right balance between the random external input, determined by *I*_*re*_ and *I*_*ratio*_, and the network generated input, determined by *esyn*_*1*_, *synweight*_1_, and **Δ**_**range**_. Producing the first, mode of the ISI histogram, which corresponds to the short ISIs of the doublets (and multiple spike short bursts), requires sufficient network input (it cannot be produced by the random external input alone). The width of this first mode is determined by the variability in network transmission (**Δ**_**range**_,). The second mode corresponds to the longer ISIs, and is mainly determined by features of the external input. The overall skew of the ISI distribution–the length of the tail–decreases with *I*_*re*_. The position of the second mode is strongly influenced by the HAP half-life (λ_*HAP*_). All of these parameters influence the spike rate, and have to be compensated against each other. Increasing *I*_*re*_ for example increases the height of the first mode which might be compensated by reducing *esyn*_*1*_. Thus fits fall within a consistent range, but are not unique. To study this we attempted multiple fits in an example “doublet-broad” cell (S2 Table), testing fits fixed by different *I*_*ratio*_ and *synweight*_*1*_. A larger *I*_*ratio*_ can be compensated by a smaller *I*_*re*_ and shorter λ_*HAP*.,_ and a larger *synweight*_*1*_ can be compensated by a smaller *esyn*_*1*_. Thus manual fitting, while not exhaustive or fully objective, gives a good understanding of how the model is working, and the essential balance between activity-dependent and -independent input signals, and intrinsic properties.

The network fits to the “doublet” cells use a neuron model with parameters in the range of the “longtail1” neurons. The “doublet broad” type fits mostly use a neuron model with parameters in the range of the “broad” neurons. However, some of the previously “doublet” classified cells do not have a second mode, and these cells (doublets19 and doublets22 in S7 Fig) can be fitted by a single neuron model.

### VMN cell types in a randomly heterogeneous network

The fits thus far establish the intrinsic properties and network connectivity required to match VMN neurons but do not demonstrate how the heterogeneous cell types might co-exist. To test the ability for matches to multiple VMN cell types to co-exist in a single model network we generated a network of 200 randomly varied model neurons, with three parameters randomly varied on a normal distribution. The neurons are based on our ‘slow HAP’ model, but include random variation in λ_*HAP*_ (mean = 40, SD = 20) sufficient to produce some cells which fall in the ‘fast HAP’ range. We also applied random variation to the input rate (*I*_re_, mean 150, SD 45) and network connectivity (*esyn*_1_, mean 0.12, SD 0.15) parameters. Neurons with an *esyn*_1_ of 0 or less do not receive any connections from the network, but can still send connections to other neurons.

The network was run for 2000 s and the resulting varied ISI distributions for each neuron are presented in S9 Fig. For comparison, in S10 Fig, we include ISI distributions for our library of recorded VMN cells, presented in the same scaling and format. The ISI distributions show matches to both the single mode, and multi-mode distributions observed in the VMN cells. The majority can be matched to “longtail”, “broad”, or “doublet-broad” type cells, but there are also examples of “doublet”, “random”, “slow DAP”, and “oscillatory” type cells. All of the model cell distributions are consistent with those observed in the VMN, including some silent cells. This a very over-simplified representation of the VMN networks where we would expect neurons to be much more structured and entrained than pure random heterogeneity, but it shows that the proposed variations in intrinsic neural properties and network connectivity are capable of explaining the range of spike patterning phenotypes observed *in vivo*.

### Network based signal generation

To begin investigating the function of such a network, we looked at the summed population spike activity of the 50 ‘slow HAP’ neurons (Fig 7), reasoning that the summed activity of a network cluster might form a signal to a downstream neuronal target population. When the network is highly connected and shows two distinct modes in the ISI histogram, spiking in individual neurons consists of short bursts. The period of these bursts is determined by the competing drives of the synaptic input rate and the duration of the HAP (which accumulates across the very short ISIs of the burst). If the network is very highly synchronised then the summed activity consists of sharp distinct peaks, but with the combined noise of random variation in synaptic input, network connections, and transmission delays these peaks become more like an oscillatory waveform. Thus, as *esyn*_*1*_ increases, the summed activity shifts from flat random activity to strong oscillating peaks, indicating that the network can function as a signal generator, turning random synaptic input into a rhythmically oscillating signal.

**Fig 8:**
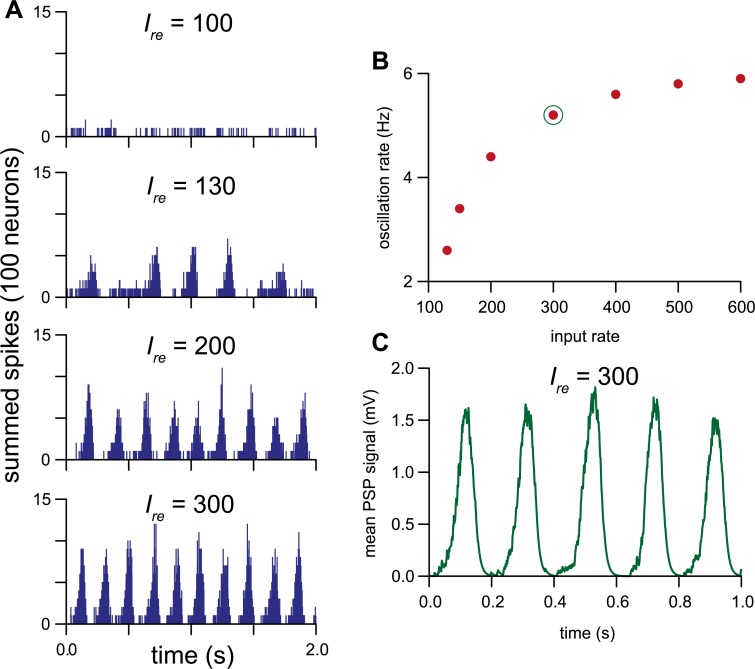
Excitatory network functioning as a signal generator. A network of 100 slow HAP neurons with *esyn*_*1*_ = 0.35 (similar to *esyn*_*1*_ = 0.7 in a 50 neuron network) tested with increasing rates of random input signal. **(A)** Summed activity of the neurons in the network, in 1-ms bins. As the input rate increases, a rhythmic population signal appears. **(B)** The rhythmic signal increases in oscillation frequency over a range from ~2.5 to 6 Hz as the input rate increases. **(C)** The green trace simulates the average postsynaptic signal generated by the neurons in the network in the condition indicated by the green circled dot in **B**.

We explored this further by testing a 100-neuron network of the same ‘slow HAP’ neurons with fixed *esyn*_*1*_ = 0.35 (equivalent to *esyn*_*1*_ = 0.7 in the 50-neuron network) with increasing rates (*I*_*re*_) of random synaptic input (Fig 8). Coherent oscillating output appears at ~2.3 Hz (*I*_*re*_ = 130). The oscillation frequency increases with the input rate, peaking at ~6 Hz (*I*_*re*_ = 600). To illustrate the collective network signal, we simulated the effect that the summed spiking activity might have on the membrane activity of a downstream neuron. This uses a reduced version of our network transmission model to model the input potential that would result from each spike generating an EPSP, essentially producing a smoothed version of the summed spike counts (Fig 8C). We further tested the scalability of the network with up to 500 neurons and produced similar results with *esyn*_1_ scaled to match the increased population, i.e. a 500 neuron network used *esyn*_1_ = 0.07 (S11 Fig). The low connection probability between two individual neurons is countered by the larger number of neurons providing a greater chance of indirect connections.

### Analysis of oscillatory spike patterning

The good fits to the “doublet” and “doublet-broad” neurons suggest that much of the more complex spike patterning observed in VMN neurons can be explained by excitatory networks of simple cells of the single ‘slow HAP’ type. However, a model network with a single simple cell type cannot explain the ISI distributions of “oscillatory” VMN cells (Fig 1). These have a multimodal ISI distribution, including modes at multiples of a period corresponding to a ‘fundamental frequency’ of 2.5–4 Hz, but also a prominent “early” mode at about 20 ms. A rhythmic signal sufficient to generate spiking on its own would produce only a single periodic mode, thus this periodic excitability suggested the idea that the oscillatory spiking activity is due to a subthreshold rhythmic signal overlaid by a random input signal. The multiple modes in the ISI histogram arise because, in any given cycle, whether or not a spike will be triggered is subject to this randomness. Experimental evidence for an underlying rhythmic signal is apparent in the average spike-triggered field potential of recorded oscillatory neurons [20].

The network of cells with a fixed slow HAP generates such a rhythm, but weakening the rhythmic signal to subthreshold by reducing network connectivity also breaks the rhythm generation, suggesting the need for heterogeneity of intrinsic neuron parameters. Initial attempts at matching the multi-modal ISI distribution using random parameter variation of slow HAP neurons produced multi-modal histograms in some slower firing cells, but these lacked the first short ISI mode. A second problem was variation between runs. If the random element in intrinsic properties was large enough to produce results different in interesting ways from that achieved with a network of identical neurons, then it also became less consistent. Only some runs produced cells which showed multi-modal histograms. Thus, a ‘slow HAP’ neuron network can produce a rhythmic signal at the expected frequency, but not the multi-modal ISI distribution. Heterogeneity is necessary, but needs to be more controlled.

**Fig 9:**
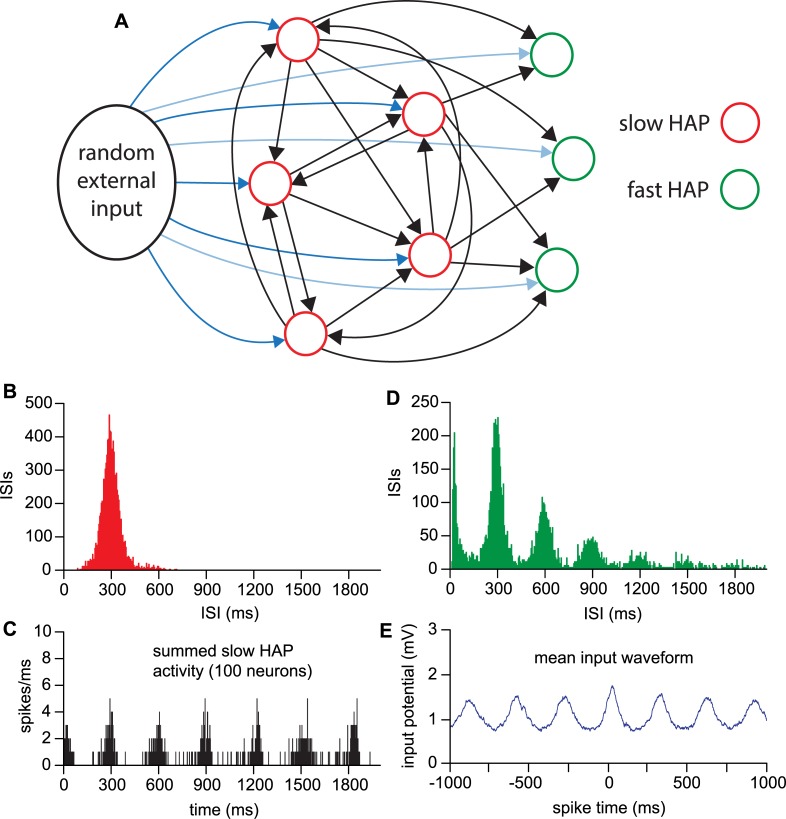
Two cell-type oscillatory network. **(A**) A network of 100 slow HAP neurons (*esyn*_*1*_ = 0.35) is extended by adding receiving fast HAP neurons (connection probability from slow HAP neurons *esyn*_*12*_ = 0.2), which also receive their own random external input. (**B)** The slow HAP neurons synchronise to generate a 3 Hz rhythmic signal; these neurons each now show a unimodal symmetrical ISI distribution with a mode corresponding to the frequency of the generated rhythm. These distributions are very like those of “regular” VMN neurons. (**C**) The summed activity of the slow HAP neurons shows that the activity of these neurons in the network is approximately synchronous. Synchrony is deliberately reduced by increasing the random element of the transmission delay (*synrange*) to produce more oscillatory summed activity. **(D**) The fast HAP neurons that receive this rhythmic input, combined with a random external input, display multimodal ISI distributions very like those of “oscillatory” neurons in the VMN. **(E**) The mean input waveform shows the mean model voltage over all spikes (at time 0) in a single fast HAP neuron, closely matching the mean waveform analysis applied to *in vivo* “oscillatory” neuron data in Fig 8A2 of [20] that showed a subthreshold 3 Hz rhythm.

Neurons which are sufficiently connected and which have a HAP slow enough to generate the oscillatory signal are also not capable of producing the early ISI mode, indicating the need for additional cells with a shorter HAP. We therefore explored a two cell-type network of ‘slow HAP’ neurons generating a rhythmic input for ‘fast HAP’ neurons, with both cell types also receiving random input (Fig 9).

### Two cell-type oscillatory network

We used an interconnected population of 100 neurons (*esyn*_*1*_ = 0.35) with a slow HAP (*k*_*HAP*_ = 60 mV, λ_HAP_ = 50 ms;) (type 1) and fed their outputs to 100 fast HAP neurons (*k*_*HAP*_ = 60 mV, λ_HAP_ = 5 ms) (type 2), parameters in Table 4. The ‘fast HAP’ neurons receive connections from the ‘slow HAP’ neurons with probability *esyn*_*12*_ = 0.2 but are not connected to each other. We also tested interconnected ‘fast HAP’ neurons, and connections from the ‘fast HAP’ neurons to the ‘slow HAP’ neurons. As these made no substantial difference to the results, we retained the simpler network, but importantly the results are not dependent on such a specific structure. To reduce the sharpness of the rhythmic peaks, compared to Fig 8, we increased the random component of the synaptic transmission delay (*synrange*) from 10 to 15 ms. The ‘slow HAP’ neurons received random input *I*_*re*_ = 200, and the ‘fast HAP’ neurons received random input *I*_*re2*_ = 80. With this, the network output was an approximately sinusoidal rhythm in the low theta range.

**Table 4:** Two cell type network model parameters (others as in Tables 1 and 3).

Name	Description	Value	Units
*vmn1*	number of type 1 neurons	100	-
*vmn2*	number of type 2 neurons	100	
*I*_re_	type 1 external input rate	180	Hz
*I*_re2_	type 2 external input rate	80	Hz
*I*_ratio_	inhibitory input ratio	0.5	-
*k*_*HAP*_	type 1 HAP amplitude per spike	60	mV
λ_*HAP*_	type 1 HAP half life	50	ms
*k*_*HAP2*_	type 2 HAP amplitude per spike	60	mV
λ_*HAP2*_	type 2 HAP half life	5	ms
*esyn*_*1*_	type 1 to type1 connection probability	0.35	-
*esyn*_*12*_	type 1 to type 2 connection probability	0.2	-
**Δ**_*range*_	transmission delay random component	15	ms

Fig 9 shows the ISI distributions for single neurons from the network of each cell type. The ISI distribution in the ‘slow HAP’ cells shows a single sharp mode at 300 ms, like the “regular” VMN neurons identified by Sabatier and Leng (2008). The fast HAP distribution shows a close match to the “oscillatory” VMN neuron of Fig 1, with a sharp early peak, followed by decaying modal peaks at 300-ms intervals. We applied the same spike waveform analysis as performed in [20] to the model’s recorded membrane potential, producing another close match to the experimental data (Fig 9).

Thus it seems that the diverse firing patterns observed in the VMN *in vivo* can all be accounted for by two intrinsic cell types receiving random external inputs and with varying degrees of random excitatory synaptic interactions between them, possibly structured into multiple sub-networks.

### Synchronous and bistable activity in VMN neurons

**Fig 10:**
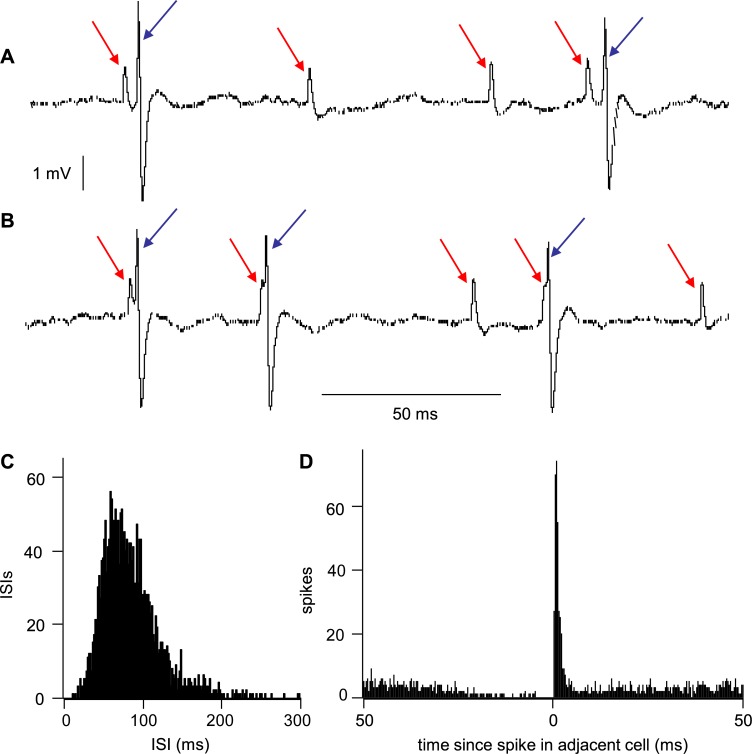
Synchronous spiking in the VMN. **(A)** Extract from the voltage trace of a recording of spike activity in the VMN (see [20]). In this recording, two cells were recorded that were clearly distinguished by spike height and waveform; the smaller spikes are arrowed in red, the larger spikes in blue. **(B)** Virtually all of the larger spikes was immediately preceded–with a slightly variable latency–by a smaller spike. **(C)** ISI distribution of the cell with the larger spikes, constructed over 300 s. This distribution is typical of “longtail1” cells in the VMN [20]. **(D**) Cross-correlation of 300 s of activity of the two cells distinguished by different spike heights–times of the smaller spike as a function of time relative to the larger spikes.

A prediction of the excitatory network model is that we should see cells *in vivo* which show a high degree of synchrony in their spiking activity. To look for evidence of this, we returned to the library of VMN neurons and inspected the original voltage recordings to find examples where, as well as the spikes from the cell analysed, smaller spikes from a second cell in the background that could be extracted by waveform analysis. From this, we found six examples of pairs of very tightly coupled cells, one of which is shown in Fig 10.

**Fig 11:**
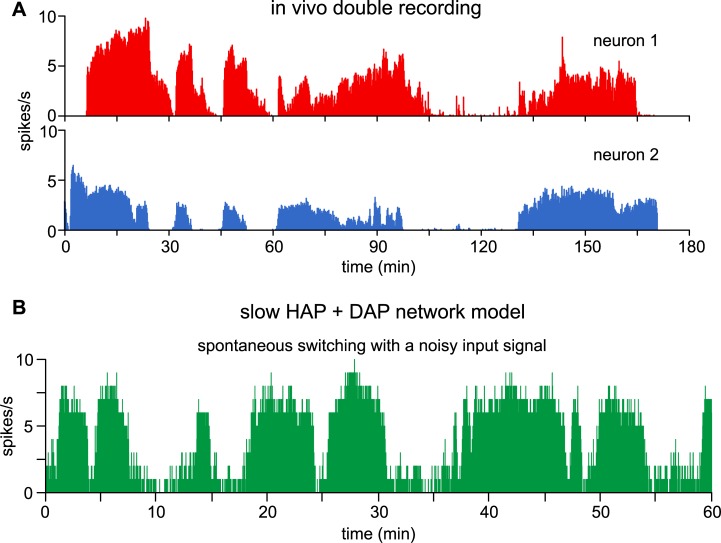
Spontaneous bistable activity in VMN neurons *in vivo* and in the model. **(A)** The double recording shows an example of two VMN neurons, recorded with a single electrode, showing loosely synchronous spiking activity with spontaneous switching between slow and fast spiking states. **(B**) An excitatory network model of slow HAP neurons with the addition to each neuron of a DAP generates bistable activity, with switching triggered by changes in external input activity. A noisy, rather than fixed *I*_*re*_ (mean *I*_*re*_ = 100) produces spontaneous switching between slow and fast spiking states, similar to the *in vivo* examples in **A**.

**Fig 12:**
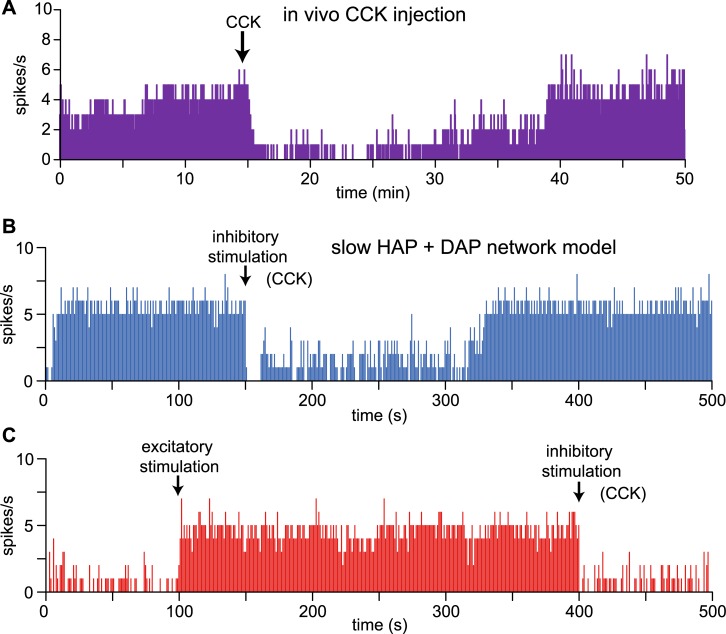
Stimulated bistable switching activity in VMN neurons *in vivo* and in the model. **(A)** Most VMN neurons are inhibited after systemic injections of CCK. In this neuron, as in many others of the VMN [27], CCK triggers the switch from a fast spiking to a slow spiking state which is maintained well beyond the duration of CCK action (systemically applied CCK disappears from plasma with a half-life of ~ 5 min). **(B)** In the same network model of Fig 11B, with a fixed input rate (*I*_*re*_ = 100), 2-s negative and positive perturbations (middle and lower panel) (*I*_*re*_ -50, *I*_*re*_ +50) were used to simulate inhibitory, and in **(C)** both stimulatory and inhibitory injected signals such as CCK or ghrelin, matching the stimulation-triggered switching between states observed *in vivo*.

Many VMN neurons *in vivo* also show bistability, switching between prolonged periods of fast and slow spiking (see Fig 9 in [20], and in one case we recorded a pair of such cells for a prolonged period, and recognised synchronous changes in activity (Fig 11A). These cells are also evidence for the VMN’s ability to act as a switching, decision-making network. Even under anaesthesia we would expect to see active decision-making mechanisms in neurons involved in regulating physiological processes; generally the homeostatic functions of hypothalamic neuronal circuits function normally under urethane anaesthesia. As well as spontaneous switching of activity, the switching can also be triggered by systemic injections of CCK [27] which mimic peripheral signals arising from the gut. CCK predominantly inhibits VMN neurons and can switch a bistable cell from stable high frequency firing to a prolonged low activity state which typically ends with an abrupt return to high-frequency firing (Fig 12A).

### Model network generated bistability

Changes to the input rate in the networks tested so far produce only gradual shifts in output spiking activity and brief input perturbations produce no sustained change in activity. Thus the strength and duration of the excitatory network connections is not sufficient for self-sustaining activity. However, adding a DAP to the neurons in a 100-neuron slow HAP network (*esyn*_*1*_ = 0.25) (parameters in Table 5) can make excitation self-sustaining. At *I*_*re*_ = 100 the network sits in a stable slow spiking state (0.85 spikes/s). At *I*_*re*_ = 103 or 104 the network switches to fast spiking state after a delay subject to the randomly timed PSPs. At *I*_*re*_ = 110 the network switches to a stable fast firing state (~6 spikes/s). For a given mean input rate, the network is typically stable in one or the other state, with no slowly decaying or accumulating element. The range of parameters which produce stability depend on a balance between input rate (*I*_*re*_), connection density (*esyn*_*1*_), and the DAP (*k*_*DAP*_ and λ_*DAP*_). A lower input rate can be compensated by a higher connection density, and a lower connection density can be compensated by a larger DAP. However, a larger DAP or higher connection density also produces a higher plateau firing rate, and so matching this to the *in vivo* examples here constrained the parameters used. Fig 11B shows an example with a noisy *I*_*re*_ (mean = 100, *tau* = 120 s, amp = 0.05) where the network randomly switches between states, like the spontaneous *in vivo* bistable activity observed experimentally and shown in Fig 11A.

**Table 5:** Bistable network model parameters (others as in Tables 1 and 3).

Name	Description	Value	Units
*vmn1*	number of type 1 neurons	100	-
*I*_re_	type 1 external input rate	100	Hz
*I*_ratio_	inhibitory input ratio	0.5	-
*k*_*HAP*_	type 1 HAP amplitude per spike	30	mV
λ_*HAP*_	type 1 HAP half life	40	ms
*k*_*DAP*_	DAP amplitude per spike	0.75	mV
λ_*DAP*_	DAP half life	750	ms
*esyn*_*1*_	type 1 to type1 connection probability	0.25	-
**Δ**_*range*_	transmission delay random component	10	ms

We failed to reproduce bistable behaviour with just a DAP, or just the excitatory connections. Thus, the bistability requires both intrinsic and network generated positive-feedback mechanisms. We tested this further by using short negative and positive perturbations to a fixed *I*_*re*_ to simulate transient inhibitory and excitatory signals (Fig 11). To match the *in vivo* experiment (Fig 12A) which shows a spontaneous return to the high state following inhibition by a CCK injection we set *I*_*re*_ = 104 (Fig 12B). To test switching by both excitatory and inhibitory pulses we set *I*_*re*_ = 100 (Fig 12C). Here short (2-s) perturbations are sufficient to switch the network between slow and fast stable states, demonstrating that the network can self-sustain both slow and fast spiking under the control of transient signals.

## Discussion

The aim of this study was to use spiking neural models to bridge between the electrophysiology of the VMN and its hypothesised function as a decision-making network. Previous studies had identified a set of neural subtypes based on patterning in recorded spike times [20]. Some of these can be fitted by a simple single neuron model, but the more complex cell types, showing patterning features such as short bursting and oscillatory activity, cannot. However, as we showed here, they can be well matched by a network of simple neuron models.

The vast majority of synaptic connections within the VMN are glutamatergic (see Introduction), and we therefore attempted to build network models using only excitatory connections. The single neuron model fits, making predictions about the intrinsic electrophysiological properties that underlie the spike patterning, divide the simpler cell types into two classes, fast HAP, and slow HAP. Using these as building blocks, we developed three types of network model which can explain the more complex spike patterning.

We showed that a network of slow HAP cells with mutual excitatory connections can generate close matches to the patterning in the “doublet” and “doublet-broad” VMN cells and mimic the short bursting activity observed in some recorded VMN neurons. Looking at the progressive changes in spike patterning with increasing network connectivity also revealed close matches to “longtail1” and “longtail2” cells, suggesting that the variation observed between these cell types may be due to different degrees of connectivity rather than varied intrinsic properties.

We have shown here that such networks can function as a signal generator, turning a random noise input into a rhythmic output in the high delta/low theta range. We showed that such a network of slow HAP cells projecting to fast HAP cells can closely match the distinctive multi-modal ISI distribution of “oscillatory” VMN neurons. Conventionally, rhythm generation in neural networks makes use of inhibitory and excitatory connections [28–31], but in this network the only inhibitory influence is intrinsic, arising from the HAP. We showed that an approximately sinusoidal waveform can be generated by a network that is deliberately not over-synchronised: weakening the synchronisation of the network by adding noise to synaptic transmission results in more wave-like summed activity, producing an oscillating signal.

These controlled examples using one or two types of homogeneous neuron models show how the more complex spike patterning observed in the VMN can arise, but leave the question of how the multiple patterning types might co-exist. The fitting of multiple recorded cells suggested that the intrinsic properties of the neurons in the VMN are highly heterogeneous. By generating a more heterogeneous network model, with variation in both intrinsic properties and network connectivity, we can produce a single network that shows matches to all of the observed VMN cell types. Producing the more simply patterned cell types requires that some cells receive less local input than others, making a prediction for the structure in the VMN. These neurons might serve as pacemakers for rhythm generation, or as a first layer that receives more of the external input signals. Less local inputs can occur either because of fewer actual connections, or because variations in input activity mean that afferent cells are silent. The library of recorded VMN neurons consists of only active cells; in fact very many VMN neurons are silent or very slow firing, such that we cannot discover their patterning.

It has been proposed that slow rhythms are important for facilitating the transfer of information between brain regions [32]. For example, if a neuron ensemble A projects to a neuron ensemble B, a subthreshold theta input from ensemble C to both A and B will, by coherently enhancing presynaptic excitability in A and postsynaptic excitability in B, selectively enhance communication from A to B. In particular, theta oscillations have been reported to synchronize the basolateral amygdala with the hippocampus and medial prefrontal cortex during periods of conditioned and innate fear in mice [33]. The amygdala has a rich reciprocal interconnection with the VMN [34] and has a common involvement in the regulation of fear, appetite and sexual behaviour [35]. Theta rhythms are also notably present in regions close to the VMN–the posterior hypothalamus and the supramamillary nucleus [36].

As summarised in the Introduction, there is evidence that the VMN is involved in decision making. A network which makes decisions in response to transient signals must be able to sustain its new state beyond the duration of that signal, while remaining responsive to new signals that might cancel that decision. An example might be feeding in a wild environment where a sensed danger would require switching from feeding behaviour to fight or flight behaviour. This requires bistable network activity. Here, we found that a network of mutually connected neurons, each with a slow HAP, and a DAP, can match the bistable activity observed in some VMN neurons. Using an excitatory network to generate bistability uses similar principles to previous work such as [37]. However, here the presence of a DAP is critical for this behaviour: neither the DAP or excitatory connections are alone sufficient. The bistability is highly robust: it is possible to generate spontaneous switching through random variations in input, but only within a very small range of input activity. Reliably triggering a switch in state requires a signal that is sustained for ~ 2 s, and the network is thus sufficiently responsive without being vulnerable to noise. By tuning the noise input signal and using transient inhibitory and excitatory perturbations to simulate injected signals such as CCK and ghrelin, this network can match the observed spontaneous, and stimulated bistable activity of VMN neurons, in particular the switching between slow and fast spiking states that has been observed *in vivo*.

Finally, we note that if two such clusters are interconnected by the sparse inhibitory neurons present in the VMN, this will generate reciprocal bistability. This suggests a natural mechanism by which the VMN can reciprocally regulate competing behavioural desires.

## Methods

### The spiking model

Single neurons are modelled using the integrate-and-fire based model previously described in [21]. For each neuron, an external input signal *I*_ext_ is generated using twin random Poisson processes to generate EPSP and IPSP counts *e*_*n*_ and *i*_*n*_ at each time step (*dt* = 1 ms in the results here), using mean rates *I*_*re*_ and *I*_*ri*_. We tested smaller time steps down to 0.1-ms to confirm that a 1-ms step was sufficiently accurate, while maintaining a practical runtime. The IPSP rate, *I*_*ri*_ is defined as *I*_*ri*_ = *I*_*ratio*_ *I*_*re*_ and the external input rate is controlled using just *I*_re_. The PSPs have fixed amplitudes *e*_*h*_ = 3 mV and *i*_*h*_ = -3 mV and are summed to give the input *I*_*ext*_:

$$I_{ext}=e_{h}e_{n}+i_{h}i_{n}$$

In the single neuron model, *I*_*ext*_ composes the entire input signal *I* such that *I* = *I*_*ext*_. This is summed to form the synaptic component of the membrane potential, *V*_*syn*_, decaying exponentially with half-life λ_*syn*_ corresponding to time constant τ_*syn*_.

$$\frac{dV_{syn}}{dt}=-\frac{V_{syn}}{\tau_{syn}}+I$$

*V*_*syn*_ is initialised to 0 mV. Time constants are calculated from half-life parameters using:

$$\tau_{x}=\frac{\lambda_{x}}{\ln(2)}$$

where *x* is the variable concerned.

The HAP variable decays exponentially with half-life parameter, λ_*HAP*_, and is incremented by *k*_*HAP*_ when a spike is fired:

$$\frac{dHAP}{dt}=-\frac{HAP}{\tau_{HAP}}+k_{HAP}\delta$$

where δ = 1 if a spike is fired at time *t*, and δ = 0 otherwise. The AHP and DAP use the same form:

$$\frac{dAHP}{dt}=-\frac{AHP}{\tau_{AHP}}+k_{AHP}\delta$$

$$\frac{dDAP}{dt}=-\frac{DAP}{\tau_{DAP}}+k_{DAP}\delta$$

At *t* = 0, the three post-spike potential variables are initialised to their respective *k* parameter values, and remain cumulative, with no post-spike reset. The voltage components are summed with the resting potential, *V*_rest_, to give the membrane potential *V*:

$$V=V_{rest}+V_{syn}-HAP-AHP+DAP$$

When *V* exceeds the spike threshold, *V*_*thresh*_, and the time since the previous spike exceeds the 2 ms absolute refractory period, a spike is fired, though its form is not modelled.

### The network model

To model a network we run multiple copies of the spiking model, calculating network input activity at each time step. Each spiking model independently generates its random external input signal. The network connections are static, and randomly generated before running the model, with each neuron connecting to each other neuron with probability *esyn*. In models with two neuron types, the connection probability is defined between each pair of types. The results here have type 1 neurons connected to each other with probability *esyn*_*1*_ and type 1 neurons connected to type 2 neurons with probability *esyn*_*12*_. At each time step (1ms) the network generated EPSPs are summed for each neuron to generate its network input signal, *I*_net_. Each connection also has a varied transmission delay component, randomly generated in a range defined by **Δ**_range._

### Network input

To model variable transmission delay, each neuron stores a 20-ms network input queue. When a neuron fires a spike, it sets a flag that it is active. At the beginning of each time step, each neuron checks for active flags on each of its connections. If a connection is active, the neuron generates a uniform random value *r*_trans_ in range 0 to 1. If *r*_trans_ > *P*(trans), where *P*(trans) is the transmission probability (in all the results here set to 0.5), then transmission is successful. The transmission delay **Δ**_trans_ is calculated as the sum of a fixed base component **Δ**_min_ and a uniform random component ranging from 0 to **Δ**_range_. **Δ**_trans_ is either fixed for each connection at network generation or generated dynamically for each transmission event. Testing both methods showed no effect on the results. **Δ**_trans_ is thus defined:

$$\Delta_{trans}=\Delta_{\min}+\Delta_{range}u_{rand}$$

where *u*_rand_ is a uniform random number between 0 and 1. The input queue is then incremented at position **Δ**_trans_. After input processing, each neuron moves its queue forward one step and the first queue position then gives the current count of network EPSPs, *n*_*n*_. The network input potentials have fixed amplitude *n*_h_ = 3 mV to give network input:

$$I_{net}=n_{h}n_{n}$$

Input *I* then becomes:

$$I=I_{ext}+I_{net}$$

### Noise input

Most of the results here use a fixed rate external input, defined by parameter *I*_*re*_. This is used to generate randomly timed EPSPs and IPSPs, producing an input signal with noise on a tens of millisecond time scale. A more noisy input signal can be generated by applying Gaussian noise to *I*_*re*_ using an Ornstein-Uhlenbeck process. *I*_*re*_ becomes a variable, defined:

$$dI_{re}=\frac{\mu_{noise}-I_{re}}{\tau_{noise}}dt+k_{noise}\sqrt{dt}g_{rand}$$

where *μ*_*noise*_ is the noise mean, *τ*_*noise*_ is the noise time course or decay rate, and *k*_*noise*_ is the noise amplitude. *g*_*rand*_ is a Gaussian (or normal) distributed random number with mean = 0 and standard deviation = 1. The variable *I*_*re*_ is initialised to *I*_*re*_ = *μ*_*noise*_.

### Spike patterning analysis and *in vivo* data

The data from the model and from experimental recordings consist of series of spike times. These are used to calculate mean firing rates and to generate ISI distributions and hazard functions, calculated from the ISI distributions as described in [38]. The ISI distributions are calculated as histograms of all the ISIs calculated from the spike times, counted in 5-ms bins. To compare data with varied spike counts, the bin counts are normalised and scaled to total 10000. The hazard function converts the absolute probabilities of the ISI distribution into conditional probabilities, so that each bin gives the chance of firing a spike in that time window (or bin), (hazard in bin [*t*, *t*+5]) = (number of intervals in bin [*t*, *t*+ 5]) / (number of intervals of length > *t*). The hazard thus shows how excitability change over time in the period following a spike.

Index of dispersion (IoD), calculated as the variance of a variable divided by its mean, is used here to measure the variation in binned spike rate across time, as previously described in [21]. Using spike times from a recorded cell or generated by the model, we count the number of spikes in successive bins. We then calculate the mean and variance of these bin counts to generate the IoD of spike rate across time. This is repeated for bin sizes of 0.5 s, 1 s, 2 s, 4 s, 6 s, 8 s, and 10 s to generate the IoD range. Purely random spike times, with no activity-dependent influence, will produce a flat IoD range.

The previously published *in vivo* spike data for model fitting are from extracellular recordings of cells in the ventromedial nucleus (VMN) of urethane-anaesthetised rats, as detailed in [20].

### Automated model fitting

The automated fitting used to fit the single neuron model to recorded cell data uses an evolutionary genetic algorithm (GA) based method, described in detail in [23]. A population of randomly generated (within specified ranges) parameter sets are run with the model and compared with the recorded cell data using a set of weighted fit measures based on the ISI distribution (divided into head range and tail range), hazard function, and IoD range, to calculate a single value fit score. The best parameter sets (the ‘parents’) are then interbred to create the next generation. The GA parameters (Table 6) include the weights for the four fit measures, and the range parameters for the ISI distribution measures. Only the ISI range parameters were altered between fits, tuned to match the ISI distribution of individual cells. We ran the GA for 40 generations each with a population size of 128. This was sufficient for the population to converge. The result is picked as the best scoring parameter set from the final generation. For each fit the GA was run 100 times, with the final fit calculated as the median parameter values of the best 10. This was repeated for each fitted cell with HAP only, HAP + AHP, and HAP + DAP.

**Table 6:** GA and fit measure parameters.

Parameter	Value
model run time	1000s
population size	128
parents	32
generations	40
mutation probability	0.05
ISI head fit weight	200
ISI tail fit weight	100
Hazard fit weight	100
IoD range fit weight	100
ISI head start	0
ISI head stop	50
ISI tail start	50
ISI tail stop	200

The GA uses a GPU based implementation with the GPU code developed in Nvidia’s CUDA [39]. A single run of the GA takes 18s running on a GeForce GTX 960 GPU.

### Implementation

The model is implemented in custom software developed in C++, compiled in Microsoft Visual Studio 2010. The graphical interface is developed in our own modelling software development toolkit, based on wxWidgets [40] and available at https://github.com/HypoModel/HypoModBase. At each 1-ms time step, the software processes input, membrane potential and spiking for each neuron in turn, using a single thread loop. A single run of a two cell type network with 200 neurons, simulated for 2000s, takes 43s. on an Intel i7-5960X processor running at 3.0GHz.

The C++ source code for the model and the GA, and a working version of the software compiled for Windows PC is available at https://github.com/HypoModel/VMNNet/releases. The code for the model is specifically in file “vmnmod.cpp”. The software archive includes all the spike data and parameter files used to generate the figures in this paper.

## Supporting information

S1 Fig Single neuron model fits to “random” cells. (EPS)

S2 Fig Single neuron model fits to “slow DAP” cells. (EPS)

S3 Fig Single neuron model fits to “longtail1” cells. (EPS)

S4 Fig Single neuron model fits to “longtail2” cells. (EPS)

S5 Fig Single neuron model fits to “broad” cells. S1–S5 Figs show the full data of which examples are shown in Fig 2, where the GA was used to produce close matches with a single neuron model to the spike patterning in five of the VMN cell types, comparing the ISI distribution, hazard function, and IoD range. The parameters are give in S1 Table.
(EPS)

S6 Fig Latency distribution between spikes of two coupled VMN cells. Detailed legend on figure.
(EPS)

S7 Fig Network model fits to “doublet” cells. (EPS)

S8 Fig Network model fits to “doublet-broad” cells. S7 and S8 Figs show network model fits to cell types which could not be fitted using the single neuron model. The “doublet” type however appears to include two subtypes, one of which does not show the second ISI mode which characterises network model generated “doublet” type patterning. The single mode subtype can be well fitted by the single neuron model. The “doublet-broad” cells all required the network model to produce a good match.
(EPS)

S9 Fig Varied ISI distributions in a heterogeneous network. Using parameters based on the ‘slow HAP’ network of Fig 7, a 200 neuron heterogeneous network was generated by applying normally distributed random variation to parameters λ_HAP_, *I*_*re*_, and esyn_1_. The 5-ms bin ISI distributions are all scaled with x-axis 0–1000 ms, and y-axis 0–500 ISIs.
(PDF)

S10 Fig ISI distributions for the VMN cell recording library. For comparison with S9 Fig, this shows ISI distributions for a library of spike pattern classified *in vivo* VMN cell recordings, from which the cells fitted in this paper were selected from. The final page shows all the cells fitted in this paper. The 5-ms bin ISI distributions are all scaled with x-axis 0–1000 ms, and y-axis 0–500 ISIs, unless otherwise stated.
(PDF)

S11 Fig Effect of *esyn*_1_ scaling against network size in a slow HAP network (matching *esyn*_1_ = 0.7 in Fig 7). (EPS)

S1 Table Single neuron model fit parameters. The best GA fit scores and parameters used to generate S1–S5 Figs.
(DOCX)

S2 Table Network model fit parameters. The parameters used to generate S7 and S8 Figs.
(DOCX)

S1 Dataset *In vivo* recorded VMN spike time data. Text files of the spike time data used in the paper.
(ZIP)
