# Supplementary figures and images for: Emergent decision-making behaviour and rhythm generation in a computational model of the ventromedial nucleus of the hypothalamus
Source: PLoS Comput Biol. 2019 Jun 3;15(6):e1007092. doi: 10.1371/journal.pcbi.1007092 (PMC6564049; doi:10.1371/journal.pcbi.1007092)

## 200 neuron heterogeneous network, 5ms bin ISI distributions

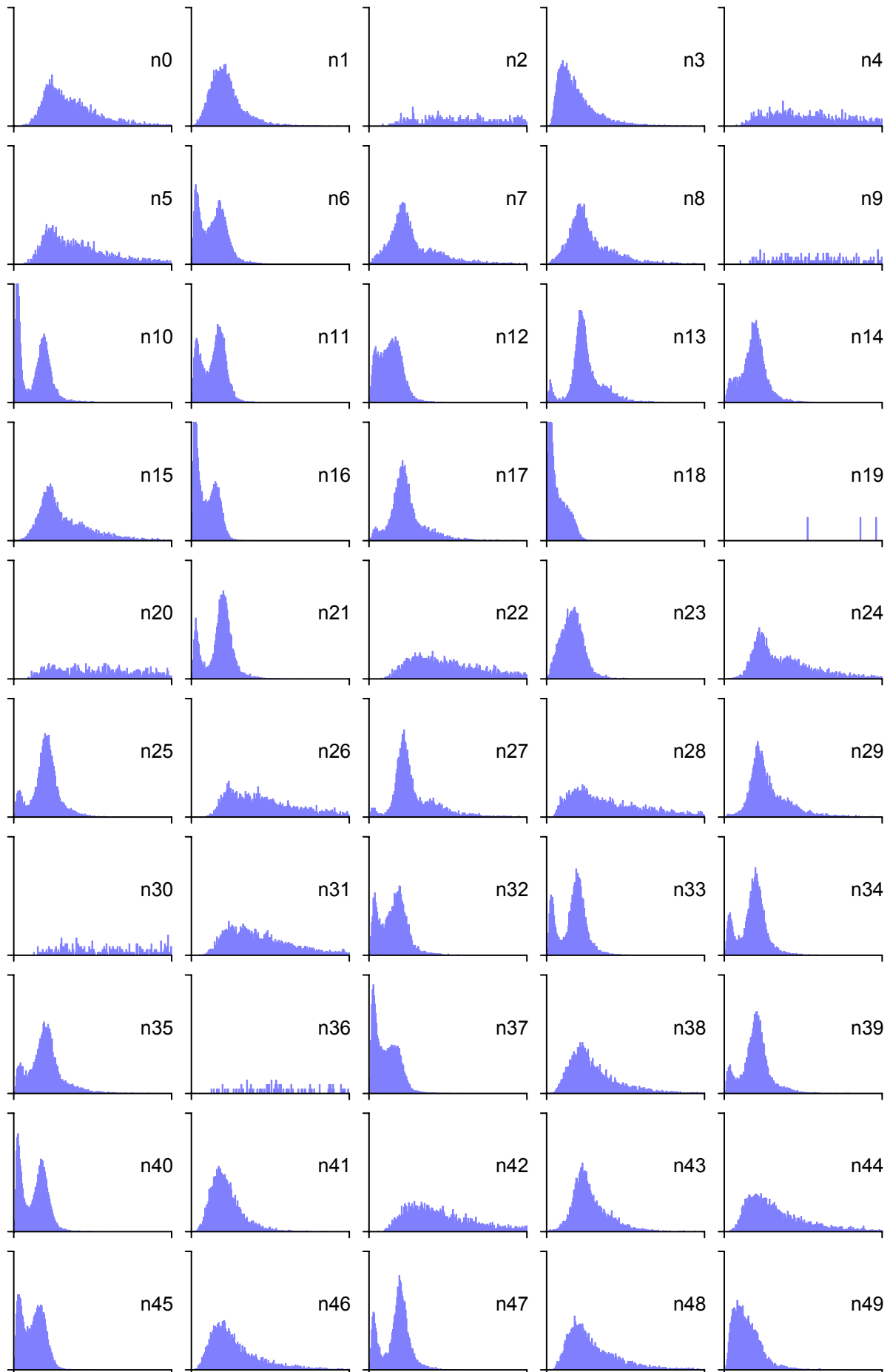

X 0 to 1000 (ms)

Y 0 to 500 (normalised ISIs)

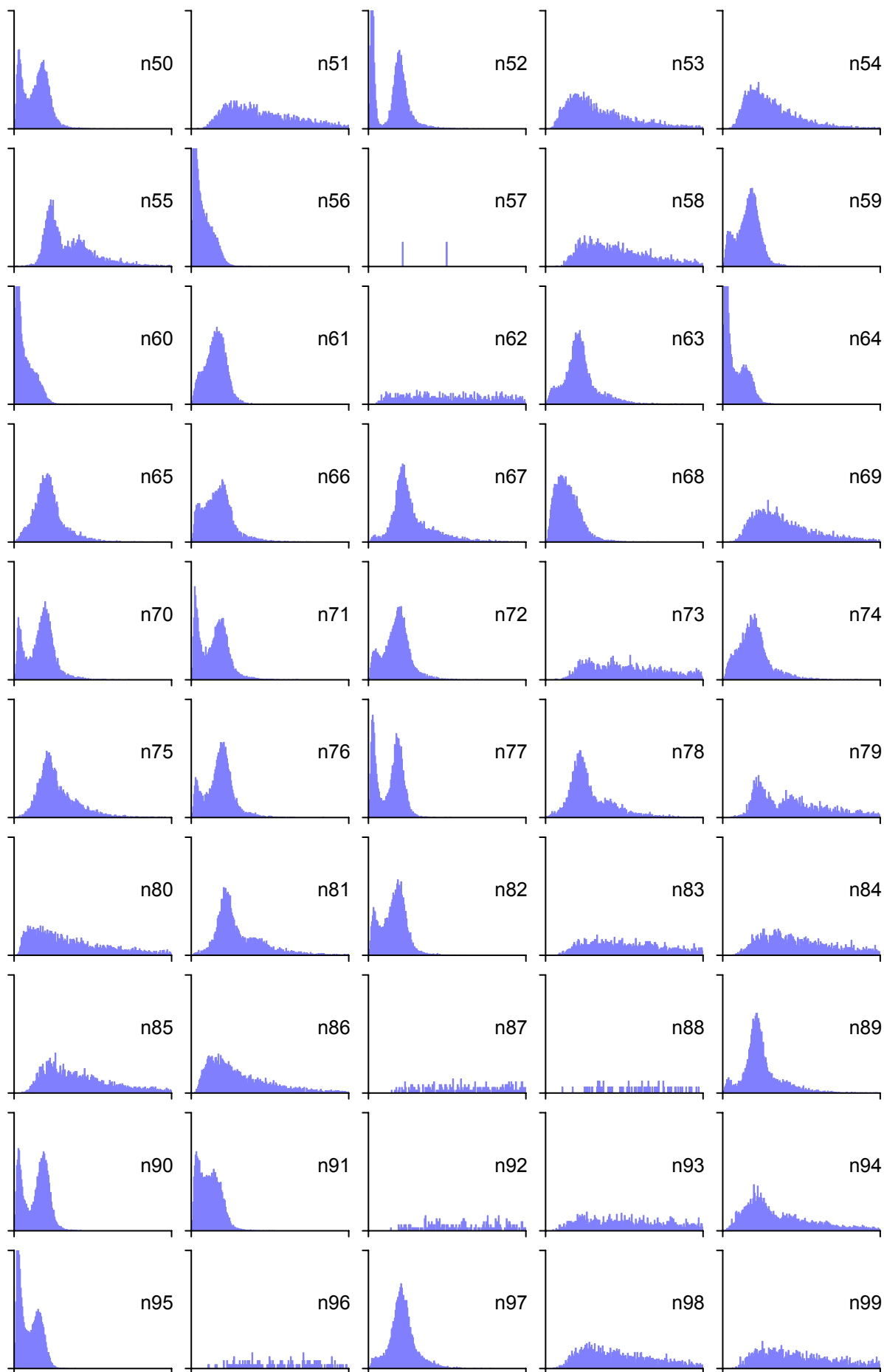

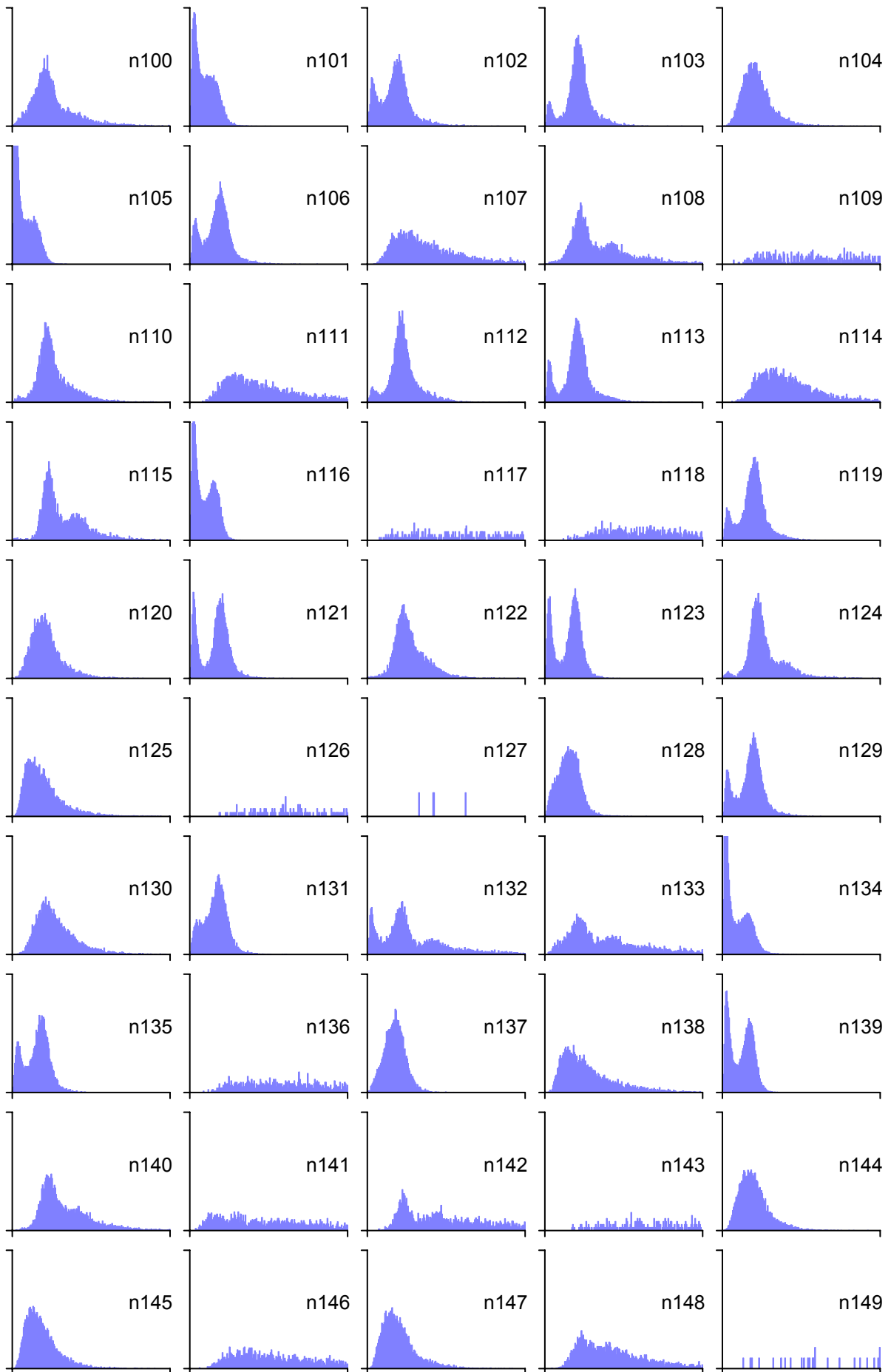

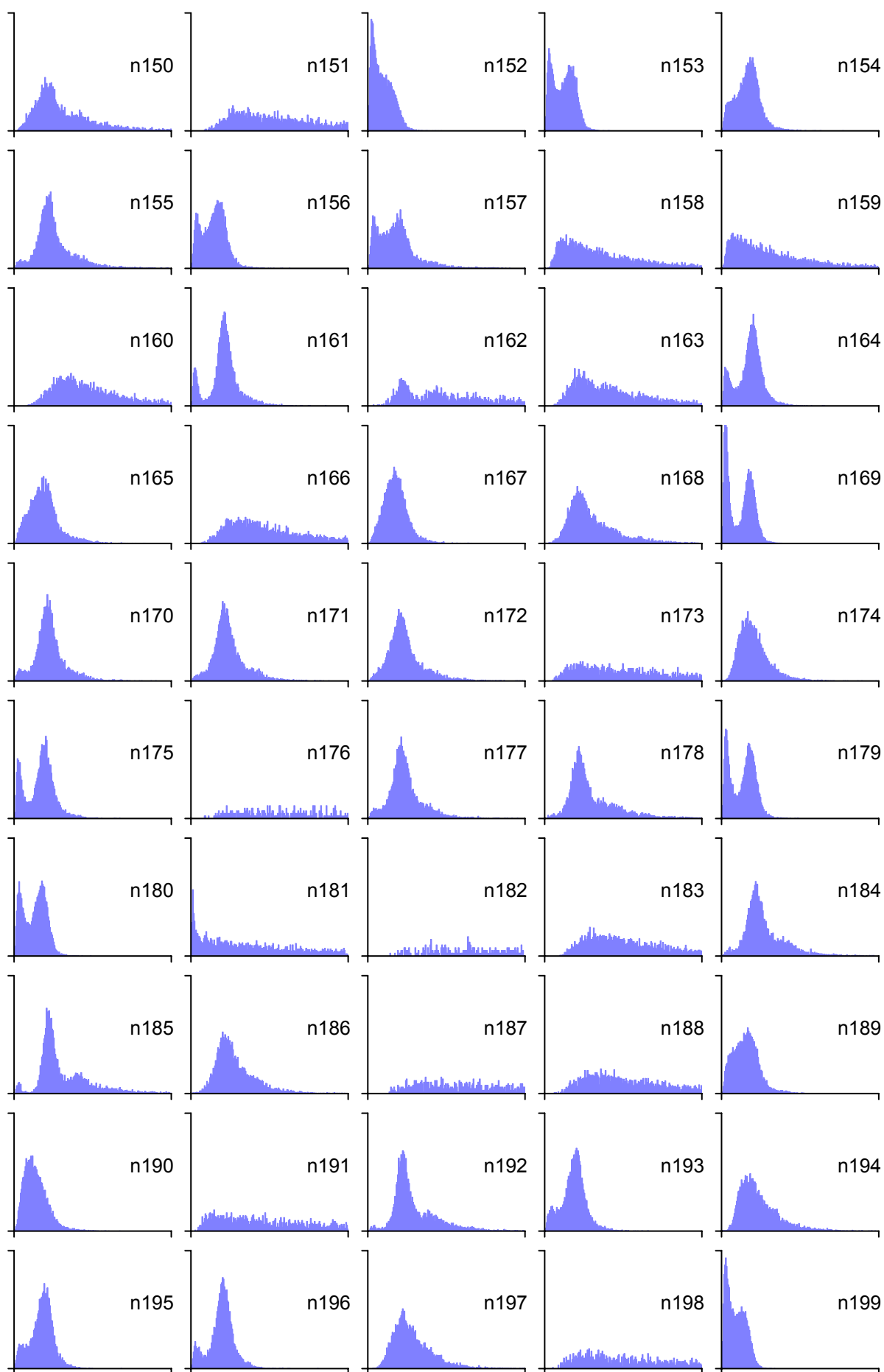

Supplement: S9 Fig — Using parameters based on the ‘slow HAP’ network of Fig 7, a 200 neuron heterogeneous network was generated by applying normally distributed random variation to parameters λHAP, Ire, and esyn1. The 5-ms bin ISI distributions are all scaled with x-axis 0–1000 ms, and y-axis 0–500 ISIs. (PDF) [file pcbi.1007092.s009.pdf]
