# Supplementary material for: Emergent decision-making behaviour and rhythm generation in a computational model of the ventromedial nucleus of the hypothalamus
Source: PLoS Comput Biol. 2019 Jun 3;15(6):e1007092. doi: 10.1371/journal.pcbi.1007092 (PMC6564049; doi:10.1371/journal.pcbi.1007092)
Supplement: S10 Fig — For comparison with S9 Fig, this shows ISI distributions for a library of spike pattern classified in vivo VMN cell recordings, from which the cells fitted in this paper were selected from. The final page shows all the cells fitted in this paper. The 5-ms bin ISI distributions are all scaled with x-axis 0–1000 ms, and y-axis 0–500 ISIs, unless otherwise stated. (PDF) [file pcbi.1007092.s010.pdf]

longtail1

5ms bin ISI histograms

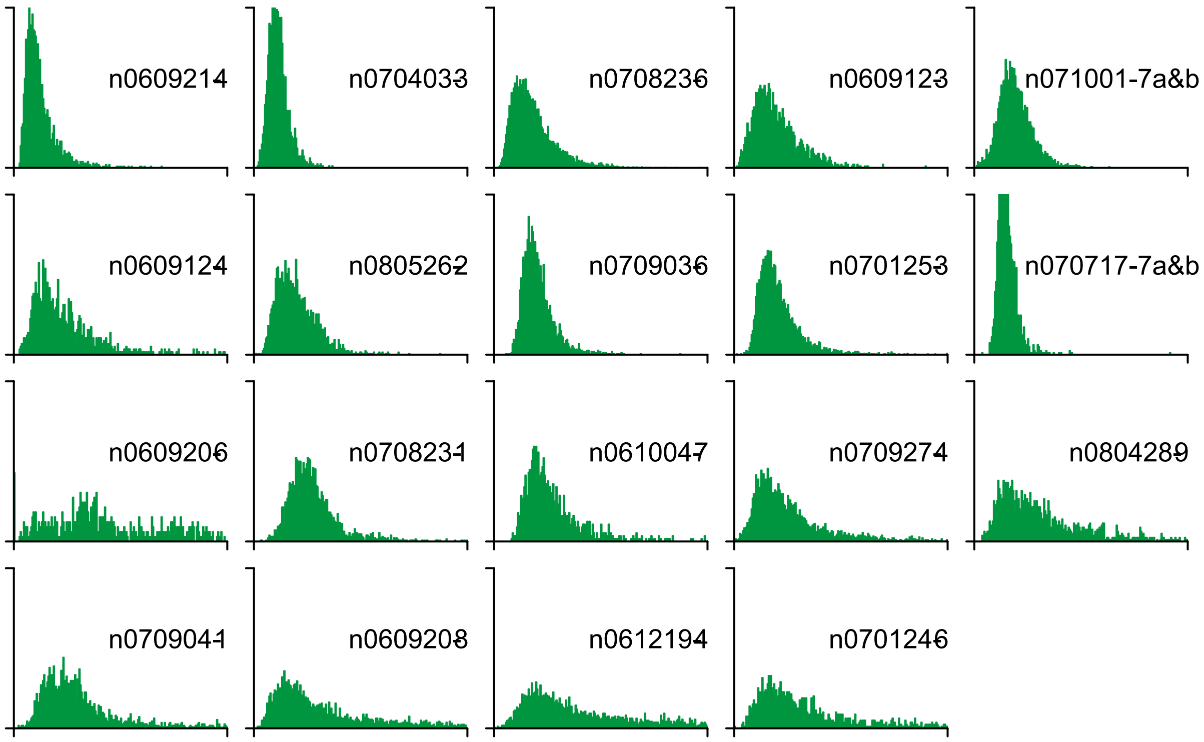

X 0 to 1000 (ms)
Y 0 to 500 (normalised ISIs)

longtail2

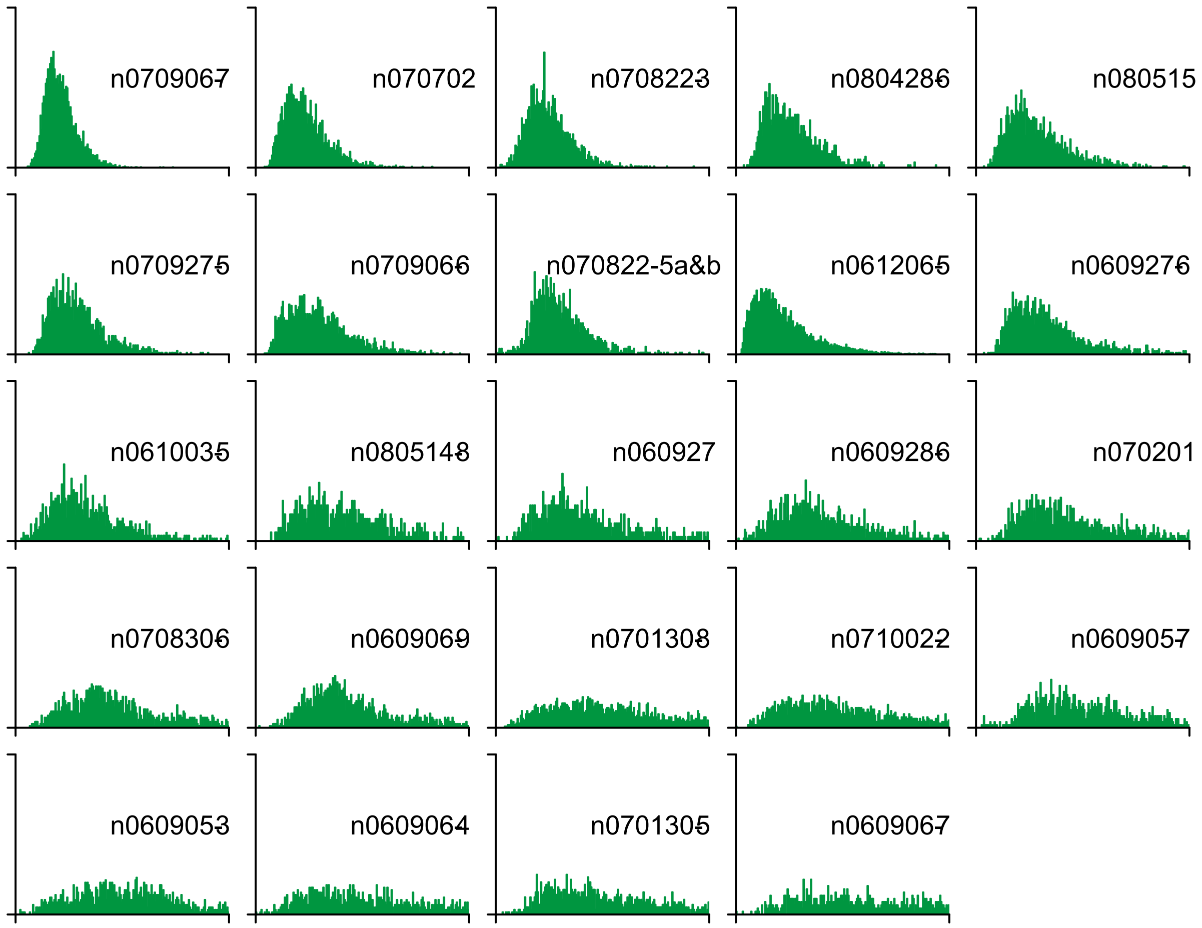
n0709067
n070702
n0708223
n0804286
n080515
n0709275
n0709066
n070822-5a&b
n0612065
n0609276
n0610035
n0805148
n060927
n0609286
n070201
n0708306
n0609069
n0701308
n0710022
n0609057
n0609053
n0609064
n0701305
n0609067

broad

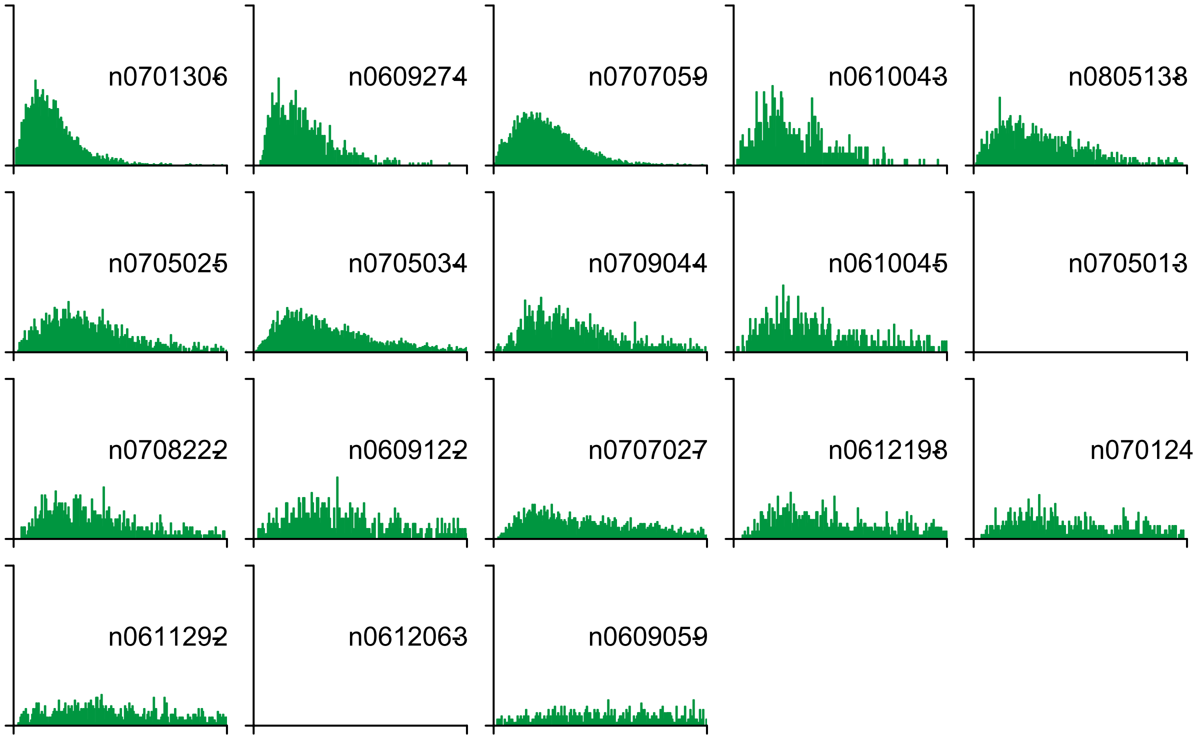
n0701306
n0609274
n0707059
n0610043
n0805138
n0705025
n0705034
n0709044
n0610045
n0705013
n0708222
n0609122
n0707027
n0612198
n070124
n0611292
n0612063
n0609059

doublets

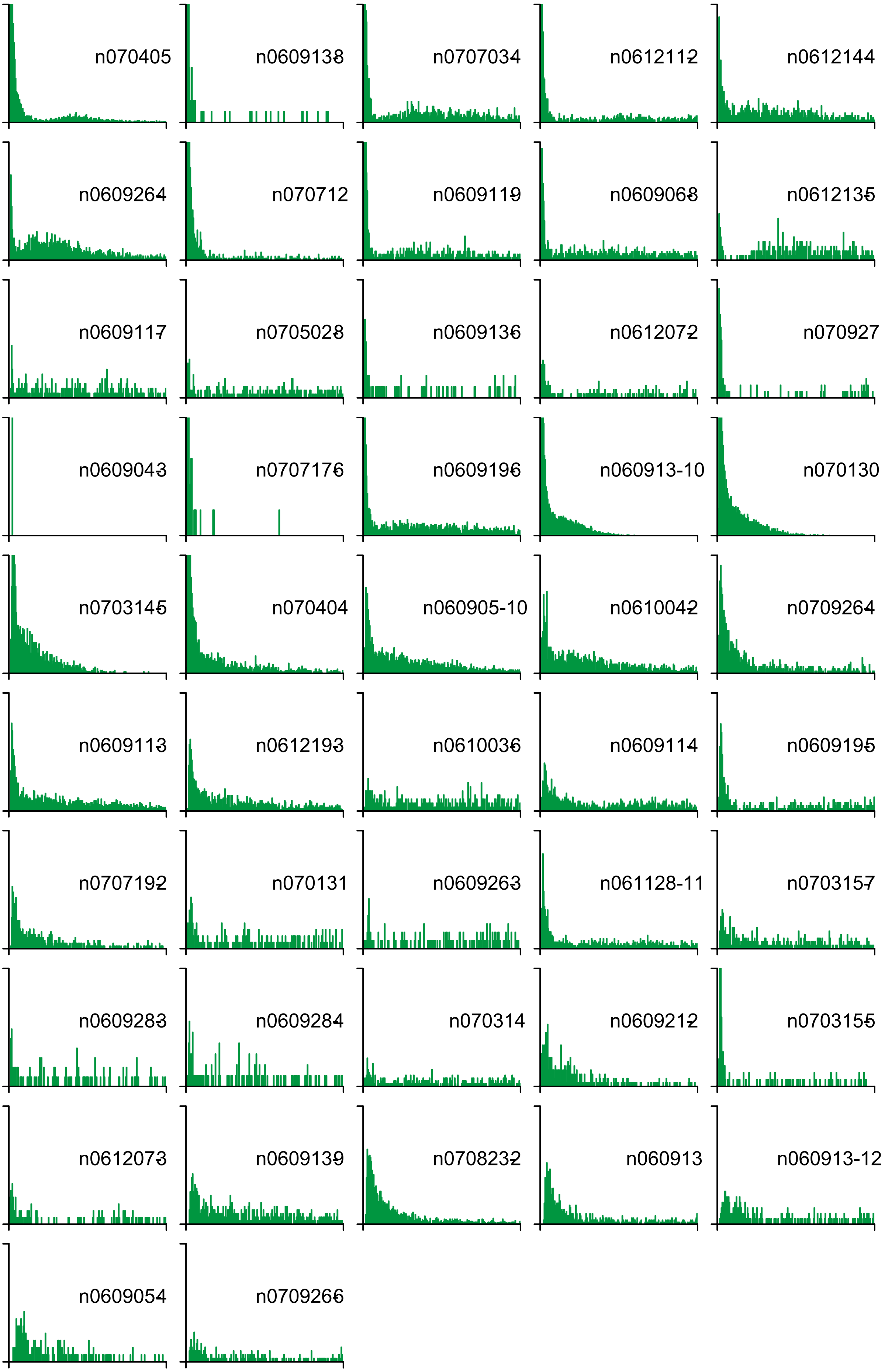
n070405
n0609138
n0707034
n0612112
n0612144
n0609264
n070712
n0609119
n0609068
n0612135
n0609117
n0705028
n0609136
n0612072
n070927
n0609043
n0707176
n0609196
n060913-10
n070130
n0703145
n070404
n060905-10
n0610042
n0709264
n0609113
n0612193
n0610036
n0609114
n0609195
n0707192
n070131
n0609263
n061128-11
n0703157
n0609283
n0609284
n070314
n0609212
n0703155
n0612073
n0609139
n0708232
n060913
n060913-12
n0609054
n0709266

doublet-broad

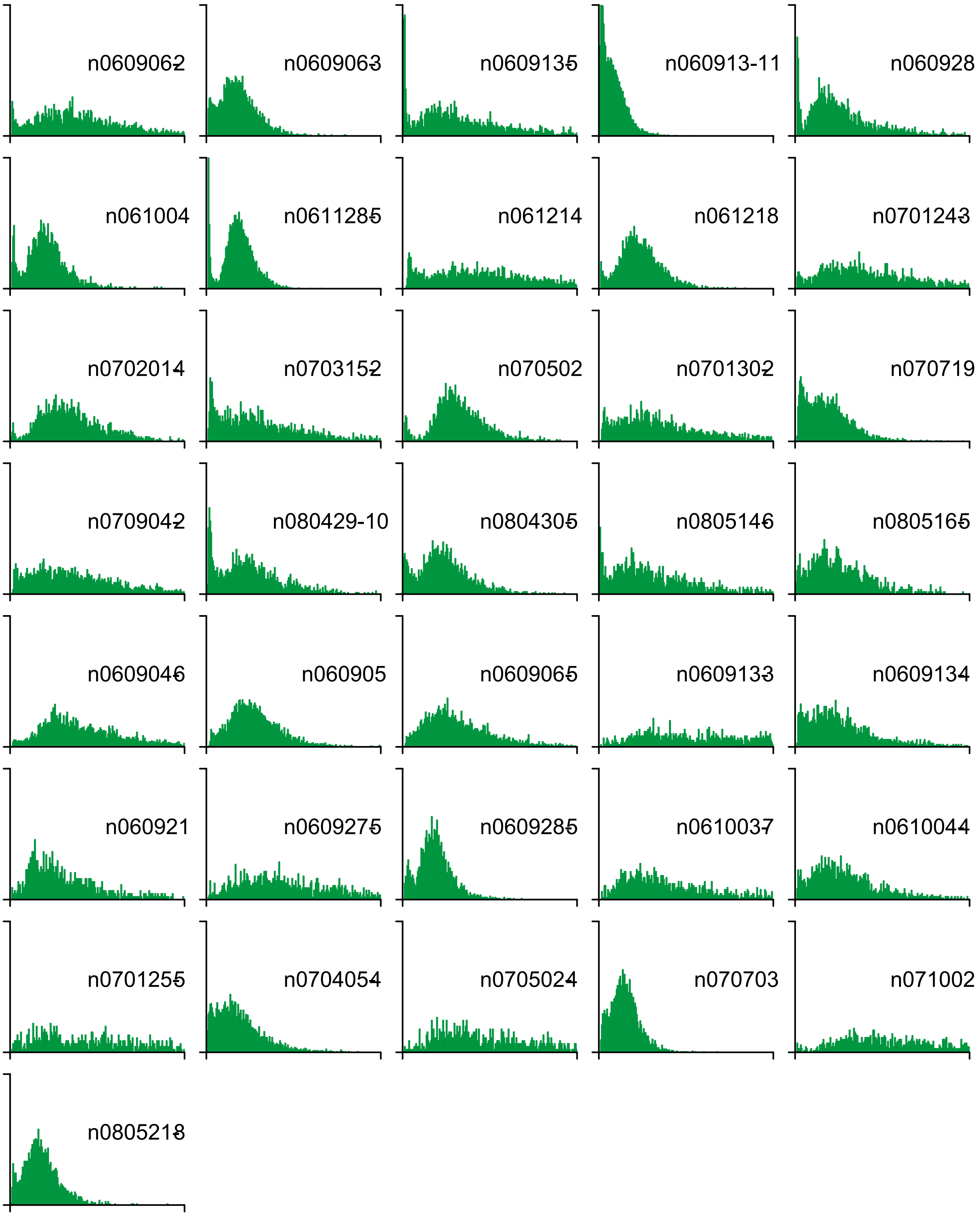

oscillatory

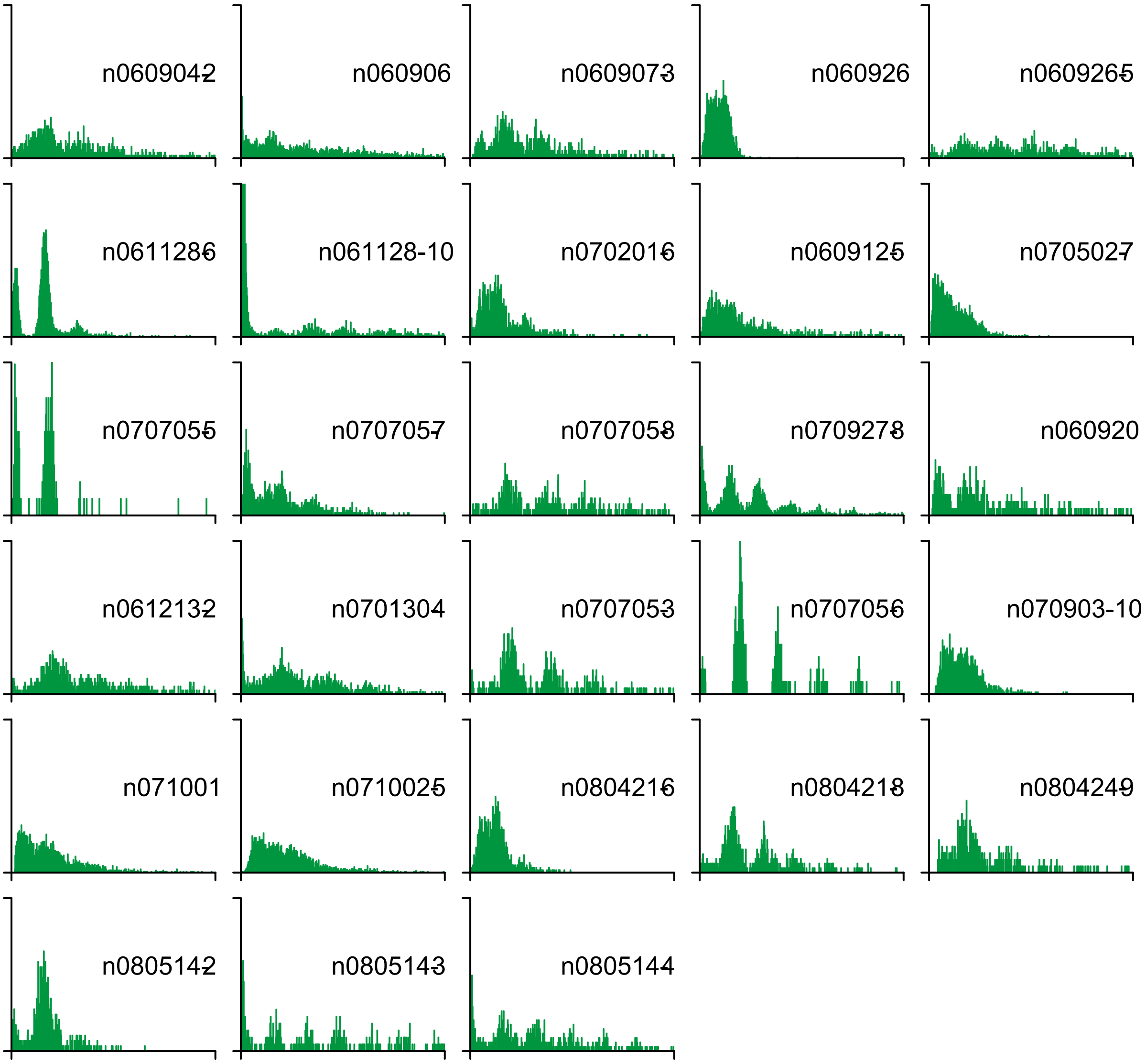

X 0 to 2000 (ms)

Y 0 to 500 (normalised ISIs)

random

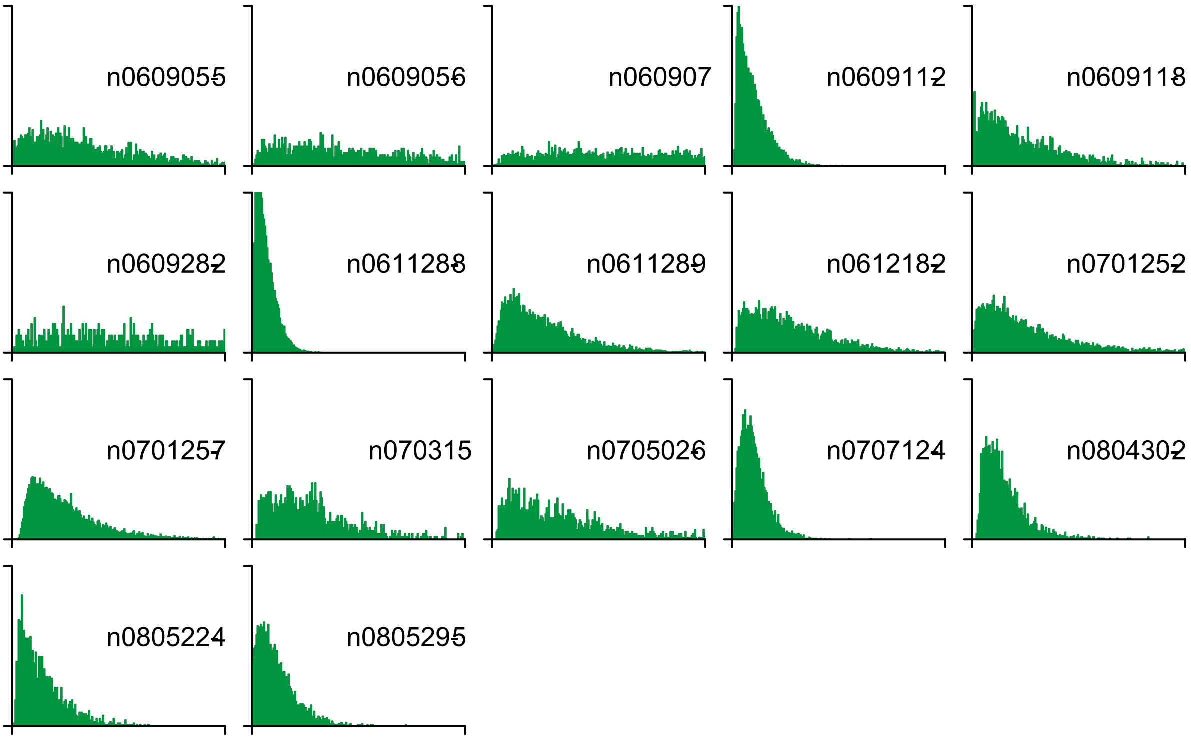
n0609055
n0609056
n060907
n0609112
n0609118
n0609282
n0611288
n0611289
n0612182
n0701252
n0701257
n070315
n0705026
n0707124
n0804302
n0805224
n0805295

slow DAP

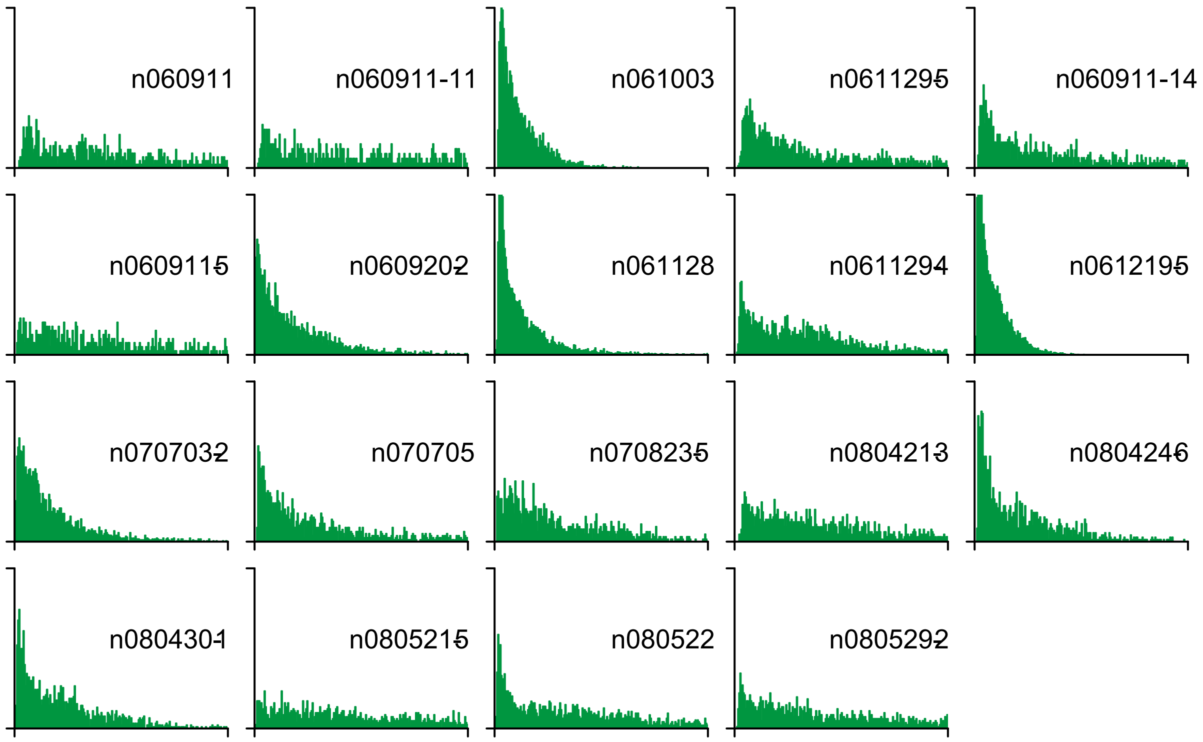

n060911
n060911-11
n061003
n061129-5
n060911-14
n060911-5
n060920-2
n061128
n061129-4
n061219-5
n070703-2
n070705
n070823-5
n080421-3
n080424-6
n080430-1
n080521-5
n080522
n080529-2

regular

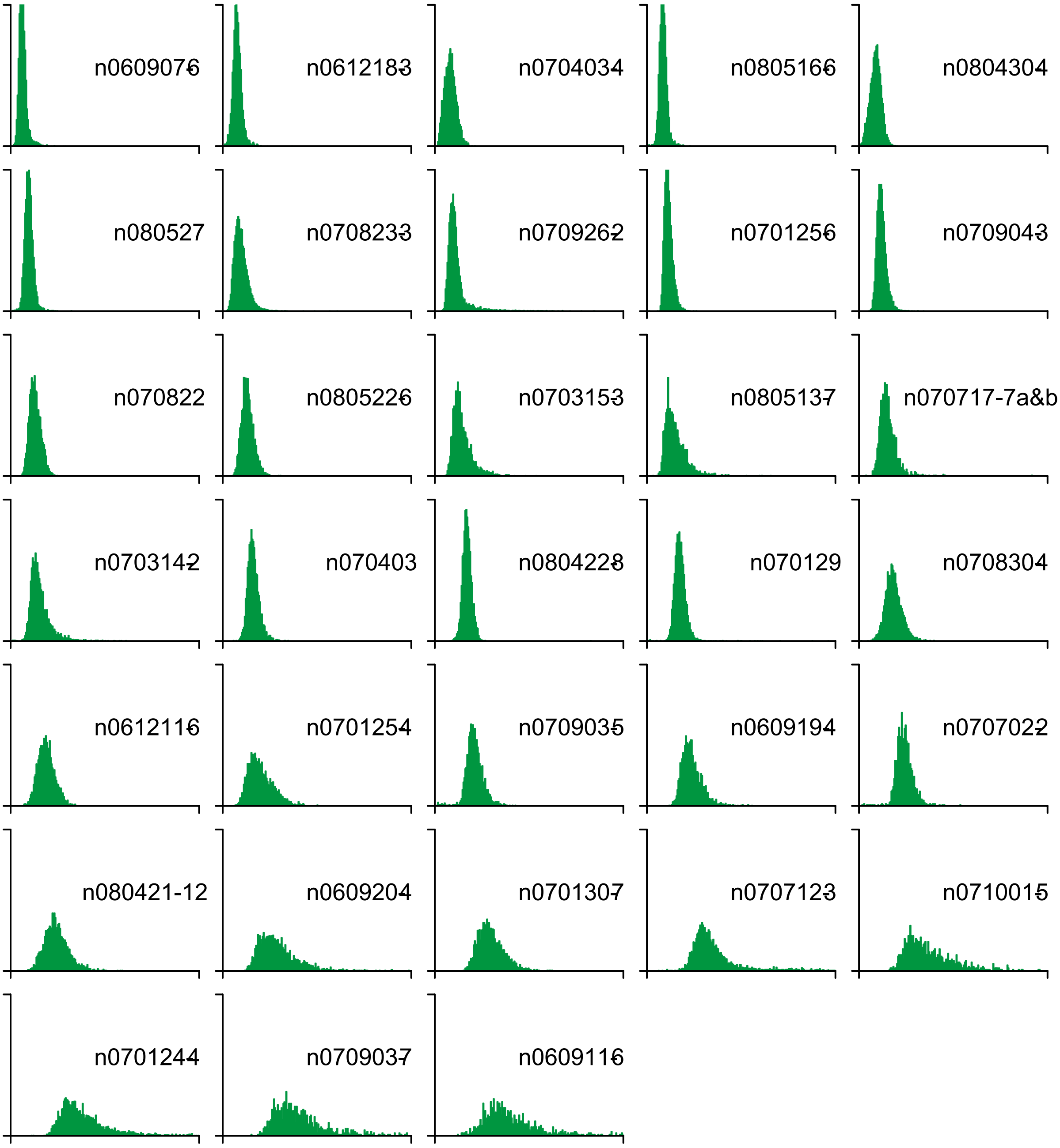

X 0 to 1000 (ms)

Y 0 to 1000 (normalised ISIs)
