# Supplementary material for: Emergent decision-making behaviour and rhythm generation in a computational model of the ventromedial nucleus of the hypothalamus
Source: PLoS Comput Biol. 2019 Jun 3;15(6):e1007092. doi: 10.1371/journal.pcbi.1007092 (PMC6564049; doi:10.1371/journal.pcbi.1007092)
Supplement: S1 Table — The best GA fit scores and parameters used to generate S1–S5 Figs. (DOCX) [file pcbi.1007092.s012.docx]

**Supplementary Table 1. Single neuron model fits to VMN cells (other parameters as Table 1)**

| **Cell** | **Best Fit** | ***I_re_*** | ***k_HAP_*** | **λ*_HAP_*** | ***k_AHP_*** | **λ*_AHP_*** | ***k_DAP_*** | **λ*_DAP_*** |
| --- | --- | --- | --- | --- | --- | --- | --- | --- |
| ran0 | 13.49 | 260.4 | 14.06 | 8.58 | 1.51 | 112.735 |  |  |
| ran3 | 5.90 | 949.5 | 67.975 | 6.045 | 1.96 | 130.655 |  |  |
| ran7 | 8.70 | 323.6 | 22.58 | 8.74 | 2.37 | 53.295 |  |  |
| ran9 | 9.86 | 235.5 | 15.3 | 9.845 |  |  | 0.14 | 1273.68 |
| ran10 | 5.80 | 344.1 | 58.655 | 12.335 | 2.01 | 70.365 |  |  |
| sd2 | 12.18 | 1672.5 | 99.635 | 6.14 | 1.86 | 471.005 |  |  |
| sd7 | 11.43 | 290.3 | 98.01 | 3.96 |  |  | 0.205 | 957.695 |
| sd8 | 14.44 | 262 | 98.12 | 4.615 |  |  |  |  |
| sd9 | 5.80 | 876.8 | 74.87 | 3.915 | 0.995 | 203.235 |  |  |
| sd17 | 11.01 | 222.9 | 27.74 | 4.645 |  |  | 1.505 | 28.93 |
| lt1-2 | 5.55 | 303 | 22.16 | 28.435 |  |  | 0.35 | 1061.945 |
| lt1-4 | 4.79 | 445.4 | 23.85 | 59.36 |  |  | 0.475 | 1256.27 |
| lt1-8 | 3.87 | 229.7 | 37.535 | 37.105 |  |  | 1.395 | 577.795 |
| lt1-11 | 9.08 | 470.1 | 45.07 | 46.46 |  |  |  |  |
| lt1-14 | 10.91 | 300.5 | 30.335 | 25.605 |  |  |  |  |
| lt2-0 | 4.69 | 309.7 | 34.235 | 53.465 |  |  | 0.685 | 1309.595 |
| lt2-1 | 6.39 | 525.6 | 26.685 | 48.115 |  |  |  |  |
| lt2-4 | 7.68 | 263.7 | 17.86 | 54.495 |  |  | 0.625 | 878.18 |
| lt2-10 | 8.28 | 242.7 | 13.53 | 67.24 |  |  | 0.58 | 921.11 |
| lt2-17 | 13.85 | 218.6 | 10.505 | 62.645 |  |  |  |  |
| broad0 | 8.47 | 428.9 | 13.325 | 27.31 |  |  |  |  |
| broad1 | 8.29 | 472.9 | 52.365 | 13.64 | 2.095 | 202.3 |  |  |
| broad2 | 9.06 | 579.7 | 11.21 | 22.25 | 4.32 | 212.16 |  |  |
| broad5 | 13.03 | 293.8 | 8.455 | 17.89 | 4.455 | 90.04 |  |  |
| broad12 | 12.88 | 245.4 | 13.005 | 29.25 | 0.63 | 691.615 |  |  |

The simpler HAP only fit was chosen as best if the HAP + AHP or HAP + DAP fit scored less than one point better on the best fit score.
