# Supplementary material for: Emergent decision-making behaviour and rhythm generation in a computational model of the ventromedial nucleus of the hypothalamus
Source: PLoS Comput Biol. 2019 Jun 3;15(6):e1007092. doi: 10.1371/journal.pcbi.1007092 (PMC6564049; doi:10.1371/journal.pcbi.1007092)
Supplement: S2 Table — The parameters used to generate S7 and S8 Figs. (DOCX) [file pcbi.1007092.s013.docx]

**Supplementary Table 2. Network model fits to VMN cells (other parameters as Tables 1 and 3)**

| **Cell** | **Neurons** | ***I_re_*** | ***I_ratio_*** | ***k_HAP_*** | **λ*_HAP_*** | ***k_DAP_*** | **λ*_DAP_*** | **esyn** | **synweight** | **∆_range_** |
| --- | --- | --- | --- | --- | --- | --- | --- | --- | --- | --- |
| d0 | 50 | 260.4 | 0.5 | 18 | 40 |  |  | 0.7 | 1.15 | 13 |
| d2 | 50 | 949.5 | 0.5 | 19 | 45 |  |  | 0.7 | 1.13 | 13 |
| d17 | 50 | 323.6 | 0.5 | 19 | 45 |  |  | 0.7 | 1.05 | 7 |
| d19 | 1 | 235.5 | 0.5 | 67.89 | 3.53 | 4.81 | 10.24 |  |  |  |
| d22 | 1 | 344.1 | 0.5 | 82.31 | 3.88 | 7.79 | 10.57 |  |  |  |
| db4 | 50 | 1672.5 | 1 | 20 | 30 |  |  | 0.5 | 1.15 | 0 |
| db8 | 50 | 290.3 | 1 | 20 | 30 |  |  | 0.4 | 1.05 | 0 |
| db12 | 50 | 210 | 1 | 25 | 50 |  |  | 0.67 | 1.0 | 0 |
| db12 | 50 | 190 | 1 | 30 | 50 |  |  | 0.4 | 1.6 | 0 |
| db12 | 50 | 140 | 0.5 | 25 | 40 |  |  | 0.55 | 1.0 |  |
| db12 | 50 | 125 | 0.5 | 25 | 40 |  |  | 0.35 | 1.6 |  |
| db17 | 50 | 876.8 | 1 | 20 | 28 |  |  | 0.4 | 1.1 | 0 |
| db27 | 50 | 222.9 | 1 | 20 | 22 | 0.1 | 150 | 0.4 | 1.02 | 0 |
